# HiC4D: forecasting spatiotemporal Hi-C data with residual ConvLSTM

**DOI:** 10.1093/bib/bbad263

**Published:** 2023-07-20

**Authors:** Tong Liu, Zheng Wang

**Affiliations:** Department of Computer Science, University of Miami, 1365 Memorial Drive, 33124, FL, USA; Department of Computer Science, University of Miami, 1365 Memorial Drive, 33124, FL, USA

**Keywords:** 4D genome, predicting spatiotemporal Hi-C data, deep learning, convolutional long short-term memory

## Abstract

The Hi-C experiments have been extensively used for the studies of genomic structures. In the last few years, spatiotemporal Hi-C has largely contributed to the investigation of genome dynamic reorganization. However, computationally modeling and forecasting spatiotemporal Hi-C data still have not been seen in the literature. We present HiC4D for dealing with the problem of forecasting spatiotemporal Hi-C data. We designed and benchmarked a novel network and named it residual ConvLSTM (ResConvLSTM), which is a combination of residual network and convolutional long short-term memory (ConvLSTM). We evaluated our new ResConvLSTM networks and compared them with the other five methods, including a naïve network (NaiveNet) that we designed as a baseline method and four outstanding video-prediction methods from the literature: ConvLSTM, spatiotemporal LSTM (ST-LSTM), self-attention LSTM (SA-LSTM) and simple video prediction (SimVP). We used eight different spatiotemporal Hi-C datasets for the blind test, including two from mouse embryogenesis, one from somatic cell nuclear transfer (SCNT) embryos, three embryogenesis datasets from different species and two non-embryogenesis datasets. Our evaluation results indicate that our ResConvLSTM networks almost always outperform the other methods on the eight blind-test datasets in terms of accurately predicting the Hi-C contact matrices at future time-steps. Our benchmarks also indicate that all of the methods that we benchmarked can successfully recover the boundaries of topologically associating domains called on the experimental Hi-C contact matrices. Taken together, our benchmarks suggest that HiC4D is an effective tool for predicting spatiotemporal Hi-C data. HiC4D is publicly available at both http://dna.cs.miami.edu/HiC4D/ and https://github.com/zwang-bioinformatics/HiC4D/.

## INTRODUCTION

Since the Hi-C technique was introduced [1], it played a vital role in identifying topologically associating domains (TADs) [2], discovering A/B compartments [1], and detecting DNA loops [3]. The genome-wide high-resolution Hi-C data were widely used in various studies, such as reconstructing chromatin three-dimensional structures [4], predicting DNA methylation [5], investigating neural development [6] and exploring Xist transcript mechanism [7].

In recent years, with the coming of the four-dimensional nucleome project [8], the dynamics of chromatin architectures during a specific nuclear or cellular process attracted much attention. Various spatiotemporal Hi-C experiments and its variants were conducted for understanding dynamic chromatin architectures in response to external stimuli [9–11] and for investigating nuclear or cellular developments, such as cardiogenesis [12], neural differentiation [6], B cell differentiation [13], B cell reprogramming [14] and embryogenesis of mouse [15–17], human [18], drosophila [19], Xenopus tropicalis [20], zebrafish [21], pig [22] and medaka [23]. These spatiotemporal Hi-C studies revealed transitions, emergences and reorganizations of chromatin architectures, and the captured spatiotemporal Hi-C data are an excellent source for exploring the relationships between gene expression and dynamics of genome reprogramming or development.

Long short-term memory (LSTM) [24] as a special recurrent neural network (RNN) for capturing long-term dependencies has been well studied and widely used in various areas, and two LSTM-variants: Gated Recurrent Unit (GRU) [25] and MUT1 [26] are attracting more and more attention in the computer vision field. The Convolutional LSTM (ConvLSTM) [27], a combination of convolution and LSTM operations, is capable of considering spatial correlations compared with fully connected LSTM (FC-LSTM). The novel architecture named spatiotemporal long short-term memory (ST-LSTM) [28, 29] included a new spatiotemporal memory state based on ConvLSTM for learning spatial and temporal representations at the same time. Self-attention LSTM (SA-LSTM) [30] introduced the popular self-attention mechanisms into ConvLSTM. Alphafold [31] used one-dimensional ConvLSTM for predicting protein structures. Hi-C-LSTM [32] used LSTM for learning low-dimensional latent representations of a Hi-C contact matrix.

RNNs based on ConvLSTMs were extensively used in video prediction. The neural networks for video prediction can be roughly split into two big categories [33]: (1) RNNs by stacking multiple ConvLSTM-based layers and (2) a spatial encoder and a spatial decoder that are usually implemented as convolutional layers together with a temporal learning part between them. The temporal-learning part in the middle may be RNN, a transformer or convolutional networks (ConvNet).

One of the newest video-prediction methods that is worth mentioning is the simple video prediction (SimVP) model, which was built completely by convolutional layers [33] and achieved state-of-the-art performance over five commonly used datasets for the task of video prediction. However, when it comes to the problem of forecasting spatiotemporal Hi-C data, we can hardly find a tool in the literature. There is a computational method 4DMax [34], which takes spatiotemporal Hi-C data as input for predicting chromatin organizations. It can interpolate Hi-C contact maps between two given time points from its predicted 4D models, but it cannot forecast spatiotemporal Hi-C at future time-steps.

In this paper, we present HiC4D for forecasting spatiotemporal Hi-C data. Since residual ConvNet (ResNet) [35] was extremely successful in building deeper networks, we newly designed and implemented a simple residual ConvLSTM and named it ResConvLSTM, which is a novel network making ConvLSTMs have more layers and with better learning abilities. In total, we benchmarked nine different methods, including ConvLSTM, ResConvLSTM and its three variants, ST-LSTM, SA-LSTM, SimVP and a naïve network. Our blind test indicates that our ResConvLSTM together with its variants almost always outperforms the other methods on eight different spatiotemporal Hi-C datasets.

## MATERIALS AND METHODS

### Spatiotemporal Hi-C datasets

**Figure 1:**
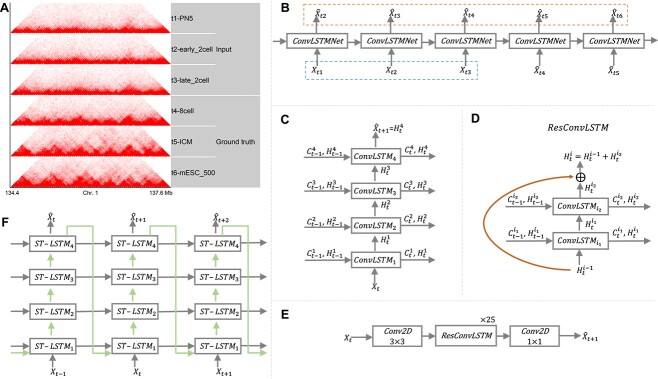
(**A**) An example of spatiotemporal Hi-C data with six time-steps from dataset 1. (**B**) The unrolled RNN architecture of a typical next-frame prediction method. The dashed blue box highlights the three time-steps as input. The contents of the dashed orange box consist of the five next-frame reconstructions. (**C**) A four-layer ConvLSTM network at time $t$. (**D**) One block of ResConvLSTM containing two ConvLSTM layers. (**E**) The architecture of ResConvLSTM network for next-frame prediction at time $t$. (**F**) A four-layer ST-LSTM network at times $t-1$, $t$ and $t+1$. The blue arrows denote the flows of spatiotemporal memory states.

We used eight different spatiotemporal Hi-C datasets in this study. These datasets were of a varying number of time-steps and read depths for each time point. The first one [15] captured Hi-C data in preimplantation embryos at the following stages: gametes (sperm and MII oocyte), pronuclear stage 5 (PN5) zygotes, early two-cell, late two-cell, eight-cell, inner cell masses (ICM) and mouse embryonic stem cells (mES). We chose the Hi-C data of the last six development stages as our first spatiotemporal Hi-C dataset (Figure 1A and Table 1). We downloaded all the valid Hi-C read pairs in the format of ‘allValidPairs’ from Gene Expression Omnibus (GEO) under accession number GSE82185. To balance sequencing depths among different time-steps, we first obtained long-range (> 20-kb) intra-chromosomal read pairs for each stage, and then downsampled read pairs to 115 million for each stage (Table S1).

**Table 1:** Overview of eight spatiotemporal Hi-C datasets.

ID	Species	NO. of time steps (we used)	System
1	Mouse	8(6)	Embryogenesis
2	Mouse	8(6)	Embryogenesis
3	Mouse	13(6)	SCNT embryos
4	Human	6(5)	Embryogenesis
5	Medaka	12(6)	Embryogenesis
6	X. tropicalis	9(6)	Embryogenesis
7	Human	5(5)	Cardiogenesis
8	Mouse	7(6)	Cell reprogramming

The second dataset [17] is very similar to the first one, and it captured Hi-C data of mouse gametes (sperm and MII oocyte) and early embryos (Table 1), including two-cell, four-cell, eight-cell, embryonic day (E)3.5 and E7.5 stages. As in dataset 1, we only used the last six stages in this study. We downloaded raw Hi-C reads from Genome Sequence Archive (GSA) under accession number PRJCA000241. We mapped the raw reads to the reference genome (mm10) using Juicer [36], used the read pair file ‘merged_nodups.txt’ to obtain long-range (> 20-kb) intra-chromosomal read pairs, and finally downsampled read pairs to 62 million for each stage (Table S2).

The third dataset [16] captured spatiotemporal Hi-C of somatic cell nuclear transfer (SCNT) embryos (Table 1). It included 13 different stages of reconstructed embryos. Considering the lower sequencing depths of some stages and being largely consistent with the time-steps of dataset 1, we only used six stages (Table S3). We downloaded the Hi-C read pairs in the format of ‘allValidPairs’ from GEO under accession number GSE146001, obtained long-range intra-chromosomal read pairs and downsampled read pairs (33 million) as above (Table S3).

The fourth dataset [18] contains six stages of spatiotemporal Hi-C during human embryogenesis, including sperm, two-cell, eight-cell, morula, blastocysts and 6-week-old embryos. We used the last five stages (Table 1) in our experiment. We downloaded the raw reads from GSA under accession number CRA000852. We mapped the raw reads to the reference genome (hg19) using Juicer [36], filtered the read pair file ‘merged_nodups.txt’ to obtain long-range (> 20-kb) intra-chromosomal read pairs, and finally downsampled read pairs to 14.5 million for each stage (Table S4).

The fifth dataset [23] captures medaka Hi-C at 12 time points before, during and after gastrulation. We selected six time points as one of our testing datasets. We downloaded raw reads from the NCBI BioProject database (PRJDB7492) and mapped them to the reference genome (GCA_002234675.1) with Juicer followed by the same filtering and down-sampling procedures (Table S5).

The sixth dataset [20] contains nine stages of spatiotemporal Hi-C captured in Xenopus tropicalis embryos. We followed the same pipeline of processing the dataset: downloading raw reads (NCBI BioProject database under accession number PRJNA606649), mapping to the reference genome (xenTro10) with Juicer and finally filtering and down-sampling (Table S6).

The last two datasets (datasets 7 and 8) were selected for benchmarking our methods on non-embryogenesis data. The developments for the two datasets are human cardiogenesis [12] and the reprogramming of mouse somatic cells into pluripotent stem cells [14], respectively. The ‘allValidPairs’ for dataset 7 were downloaded from GEO, GSE106690, and the details for filtering and down-sampling procedures can be found in Table S7. The raw reads for dataset 8 were obtained from GEO GSE96611 and mapped to the reference genome (hg38) with Juicer followed by the same filtering and down-sampling steps (Table S8).

To reduce the effects from different numbers of valid read pairs, we set a parameter (maxHiC) for each dataset based on their total number of read pairs after down-sampling, chromosome numbers and chromosome lengths to rescale Hi-C contacts to the range [0, 1].

### HiC4D overview

The training data were extracted from dataset 1. From all chromosomes from 1 to X, we extracted validation data on chromosome 19, two chromosomes (i.e. 2 and 6) were left for the blind test and the rest chromosomes were used for generating training data. The other seven datasets were selected for blind testing: we tested datasets 2–3 on two chromosomes (2 and 6) and the rest datasets (4–8) on all chromosomes. Considering read depths, we only focused on the resolution of 40 kb in this study. The samples for each chromosome were generated along the diagonal of its 2D raw contact matrix with a sliding window of size $50\times 50$ and a step of size 3 bins. The samples were concatenated as an $n\times{t}\times 1\times 50\times 50$ five-dimensional tensor, where n is the total number of samples and t is the number of time-steps. The main purpose of this study is to use the Hi-C data of the first three time-steps ($t_{1}$, $t_{2}$ and $t_{3}$) as input to predict the corresponding Hi-C data of the last three time-steps ($t_{4}$, $t_{5}$ and $t_{6}$).

We tested two types of frame prediction methods: next-frame and three-step ahead. The next-frame methods consist of ConvLSTM, our newly designed ResConvLSTM, its variants (ResConvGRU, ResConvMUT and ResConvLSTM2) and two novel ConvLSTM-based methods (ST-LSTM and SA-LSTM). The three-step ahead methods include SimVP and NaiveNet. These nine methods were trained with the same data. The best models for the blind test were the ones that achieved the best performance on validation data. Since one pixel may be predicted more than one time, its final prediction is the average value of all predictions.

After obtaining predictions for each testing chromosome at the future time-steps, we evaluated each method mainly by quantifying the similarity scores between ground truth and predictions for each time-step.

### Next-frame method

The input of next-frame methods $X_{t}$ at time $t$ is an $n\times 1\times 50\times 50$ four-dimensional tensor. After passing through the network, we obtain the output $\hat{X}_{t+1}$, which is thought of as the reconstruction of $X_{t+1}$ (Figure 1B). Since our input time-steps are $t_{1}$, $t_{2}$ and $t_{3}$, the fourth and the following $X_{t}$ are directly from the output of their previous time-step (Figure 1B). The loss is calculated between $X_{t}$ and reconstructed $\hat{X}_{t}$, where $t$ is from two to six. Technical details of the next-frame methods (ConvLSTM, ResConvLSTM and ST-LSTM) will be presented in the following three subsections.

#### Convolutional LSTMs

The first ConvLSTM (ConvLSTM-1) we implemented and benchmarked in this study is the same as the one published in the paper that first applied convolutional operation to LSTM [27]. The following equations describe a typical $l$-th ConvLSTM-1 layer at time $t$ with the following three inputs: the input data $X_{t}$, the last memory cell state $C^{l}_{t-1}$ and the last hidden state $H_{t-1}^{l}$: 


\begin{align*} i_{t} &=\sigma\left(W_{xi}\ast X_{t}+W_{hi}\ast H_{t-1}^{l}+W_{ci}\circ C_{t-1}^{l}+b_{i}\right) \nonumber \\ f_{t} &=\sigma\left(W_{xf}\ast X_{t}+W_{hf}\ast H_{t-1}^{l}+W_{cf}\circ C_{t-1}^{l}+b_{f}\right) \nonumber \\ C_{t}^{l} &=f_{t}\circ C_{t-1}^{l}+i_{t}\circ\tanh{\left(W_{xc}\ast X_{t}+W_{hc}\ast H_{t-1}^{l}+b_{c}\right)} \nonumber \\ o_{t} &=\sigma(W_{xo}\ast X_{t}+W_{ho}\ast H_{t-1}^{l}+W_{co}\circ C_{t}^{l}+b_{o}) \nonumber \\ H_{t}^{l} &=o_{t}\circ\tanh(C_{t}^{l}), \nonumber \end{align*}

where $\ast $ denotes the convolution operator, $\sigma $ denotes the sigmoid function, $\circ $ denotes the Hadamard product and $W$ and $b$ are the weight and bias parameters that need to be learned. Since there are various LSTM variants, we added two more ConvLSTMs (i.e. ConvLSTM-2 and ConvLSTM-3). When calculating $i_{t}$, $f_{t}$ and $o_{t}$, we removed the Hadamard products in ConvLSTM-2 and replaced all the Hadamard products with convolutional operations in ConvLSTM-3 (see supplementary materials for details). The other parts for the three ConvLSTMs remain the same. We only used one of them for the blind test, and the evaluation results for the three ConvLSTM variants are shown in the Results section. We built the ConvLSTM networks by simply stacking multiple ConvLSTM layers (Figure 1C).

#### ResConvLSTM and its three variants

We designed ResConvLSTM, which is a combination of residual ConvNet and ConvLSTM (Figure 1D) and is similar to previous residual LSTMs [37, 38]. The $i$-th ResConvLSTM block shown in Figure 1D contains two ConvLSTM-1 layers (i.e. $ConvLSTM_{i_{1}}$ and $ConvLSTM_{i_{2}}$). The final output $H_{t}^{i}$ of this block at time $t$ is equal to 


\begin{align*}& H_{t}^{i}=H_{t}^{i-1}+H_{t}^{i_{2}}, \end{align*}

where the input $H_{t}^{i-1}$ is the output of the previous ResConvLSTM block and the updated hidden state $H_{t}^{i_{2}}$ is the output of $ConvLSTM_{i_{2}}$. The skip connection can make the output $H_{t}^{i}$ directly link to the input $H_{t}^{i-1}$. The final ResConvLSTM network we used for the blind test is shown in Figure 1E. It contains two 3$\times $3 2D convolutional layers for increasing and reducing hidden channels, and the middle part is built by stacking 25 ResConvLSTM blocks.

We replaced all ConvLSTMs in the ResConvLSTM network with convolutional GRU and MUT1 (see supplementary materials for details) and obtained two more Residual RNN networks (ResConvGRU and ResConvMUT). The two RNNs (GRU and MUT) outperform LSTM on some tasks [26]. Therefore, we think it is worth benchmarking the two methods together with residual networks. We also extended ResConvLSTM by concatenating outputs from every five ResConvLSTM blocks as the final input of the last output layer (ResConvLSTM2 in Figure S1), which was inspired by our previous work [39].

#### ST-LSTM and SA-LSTM

The ST-LSTM introduced a novel spatiotemporal memory state [28, 29]. It can flow in both bottom-up and top-down directions, whereas the memory cell state and the hidden state can only flow in horizontal and bottom-up directions, respectively. The following equations describe an ST-LSTM layer at time $t$: 


\begin{align*} i_{t} &=\sigma\left(W_{xi}{\ast X}_{t}+W_{hi}\ast H_{t-1}^{l}{+\ b}_{i}\right) \\ f_{t} &=\sigma\left(W_{xf}\ast X_{t}+W_{hf}\ast H_{t-1}^{l}+b_{f}\right) \\ C_{t}^{l} &=f_{t}\circ C_{t-1}^{l}+i_{t}\circ\tanh{\left(W_{xc}{\ast X}_{t}+W_{hc}\ast H_{t-1}^{l}+b_{c}\right)} \\ i_{t}^{\prime} &=\sigma\left(W_{xi}^{\prime}{\ast}{{X}}_{t} +W_{mi}\ast M_{t}^{l-1}+b_{i}^{\prime}\right) \\ f_{t}^{\prime} &=\sigma\left(W_{xf}^{\prime}\ast X_{t}+W_{mf}\ast M_{t}^{l-1}+b_{f}^{\prime}\right) \\ M_{t}^{l} &=f_{t}^{\prime}\circ M_{t}^{l-1}+i_{t}^{\prime}\circ\tanh{\left(W_{xm}{\ast X}_{t}+W_{mm}\ast M_{t}^{l-1}+b_{m}\right)} \\ o_{t} &=\sigma(W_{xo}\ast X_{t}+W_{ho}\ast H_{t-1}^{l}+W_{co}\ast C_{t}^{l}+W_{mo}{\ast M}_{t}^{l}+b_{o}) \\ H_{t}^{l} &=o_{t}\circ\tanh(W_{1}\ast\left[C_{t}^{l},\ M_{t}^{l}\right]), \end{align*}

where $M_{t}^{l}$ is the spatiotemporal memory state for the $l$-th layer at time $t$ and $W_{1}$ is 1$\times $1 2D convolutional operation for reducing the hidden dimension. The process of generating $M_{t}^{l}$ is very similar to deriving $C_{t}^{l}$. We implemented the final ST-LSTM networks by stacking multiple ST-LSTM layers (Figure 1F).

We also implemented an SA-LSTM [30], which is very similar to ConvLSTM. The main difference is that it preprocessed the input hidden state in each ConvLSTM with a self-attention module.

### The three-step ahead methods: SimVP and NaiveNet

As its name suggests, the three-step ahead methods mean that we predict three future frames in one prediction. We used the same network of SimVP as described in [33]. The batch size and the hidden dimension were set to 32 and 128, respectively. The other hyperparameters were with defaults. We designed a naïve 3D convolutional neural network (NaiveNet) as a baseline. The NaiveNet (Figure S2) contains three 3D convolutional layers. Each of the first two layers is followed by Group normalization (number of groups set to 2) [40] and LeakyReLU (negative slope set to 0.2).

The model of NaiveNet for the blind test was trained with the following hyperparameters: batch size 32, hidden dimension 128 and kernel sizes 7 for the two spatial dimensions (height and width) and 1 for the temporal dimension. The input of three-step ahead methods is an $n\times 3\times 1\times 50\times 50$ five-dimensional tensor, where 3 denotes the first three time-steps (i.e. $t_{1}$, $t_{2}$ and $t_{3}$). The output is still an $n\times 3\times 1\times 50\times 50$ five-dimensional tensor, but here 3 denotes the last three time-steps (i.e. $t_{4}$, $t_{5}$ and $t_{6}$). The loss is calculated between the output and the ground truth at the last three time-steps.

### Implementation details

All models were implemented in PyTorch [41]. Unless otherwise stated, the loss function for all the models is mean squared error (MSE), and the optimizer for training all models is Adam [42] with a learning rate set to 0.0001. All models were trained on an NVIDIA A100 GPU with 40 GB memory.

### Evaluation metrics

The main evaluation strategy is by quantifying similarity or reproducibility between ground-truth and predicted Hi-C contact matrices at the future time-steps. The two metrics we used are Pearson correlation coefficients at each genomic distance (10–30 bins) and stratum-adjusted correlation coefficient (SCC) from HiCRep [43]. When running HiCRep, we set the smoothing parameter to 5 and the lower and upper bounds of the genomic distance to 400 000 and 1600 000, respectively. We also calculated insulation scores [44] (see supplementary materials for details) for evaluating the ability to recover TAD boundaries.

## RESULTS

### Hyperparameter tuning and model selection

For training ConvLSTM-based models, we tested various hyperparameter combinations (see Table S9). For the three ConvLSTM networks (ConvLSTM-1, ConvLSTM-2 and ConvLSTM-3), the first two achieved a smaller MSE loss (0.0067 and 0.00669) than the last one (0.00684). To select one for the blind test, we further evaluated the three networks using Pearson correlations on chromosome 19 at each genomic distance (Figure S3). In general, ConvLSTM-1 performs better than the other two. Therefore, the four-layer ConvLSTM-1 was selected as the representative of ConvLSTM networks for the blind test.

For tuning ResConvLSTM networks, we found three hyperparameter combinations (see Table S9) achieved almost the same best losses (0.00666, 0.00664 and 0.00666). Therefore, we further evaluated the three models using Pearson correlations (Figure S4) and observed that deeper ResConvLSTM networks perform better, especially at time $t_{6}$. The model with 25 ResConvLSTM blocks (52 layers) was used for the blind test. Its three variants (ResConvGRU, ResConvMUT and ResConvLSTM2) were trained with the same hyperparameters as ResConvLSTM.

For tuning ST-LSTM networks, we trained two more models with the loss function equal to $MSE+0.1\times decouple$ [29]. The further evaluation results for the model with the smallest MSE loss and the two more models are shown in Figure S5, indicating that adding decouple loss during training does not provide better performance. Therefore, the four-layer ST-LSTM model with the smallest MSE loss was selected for the blind test. Since SA-LSTM consumes high memory, we set a relatively small hidden dimension for the self-attention module (Table S9). The final SA-LSTM model that we used was the one that achieved the smallest validation loss.

We also reported the MSE losses of two three-step ahead methods (SimVP and NaiveNet) in Table S9. It is obvious that their losses are larger than those of next-frame methods because the latter also consider the reconstruction errors of times $t_{2}$ and $t_{3}$, which are usually smaller because spatiotemporal Hi-C at times $t_{2}$ and $t_{3}$ are easier to learn. Taken together, all the benchmarking models for the blind test were trained with the same batch size of 32 and the same hidden dimension of 128 (32 for ResConvLSTM and its three variants because of GPU memory limitation). In addition, we also borrowed the wide [45] and dense [46] concepts from convolutional neural networks onto ConvLSTMs but did not obtain a smaller validation loss (data and details not shown).

### Benchmarks on dataset 1

**Figure 2:**
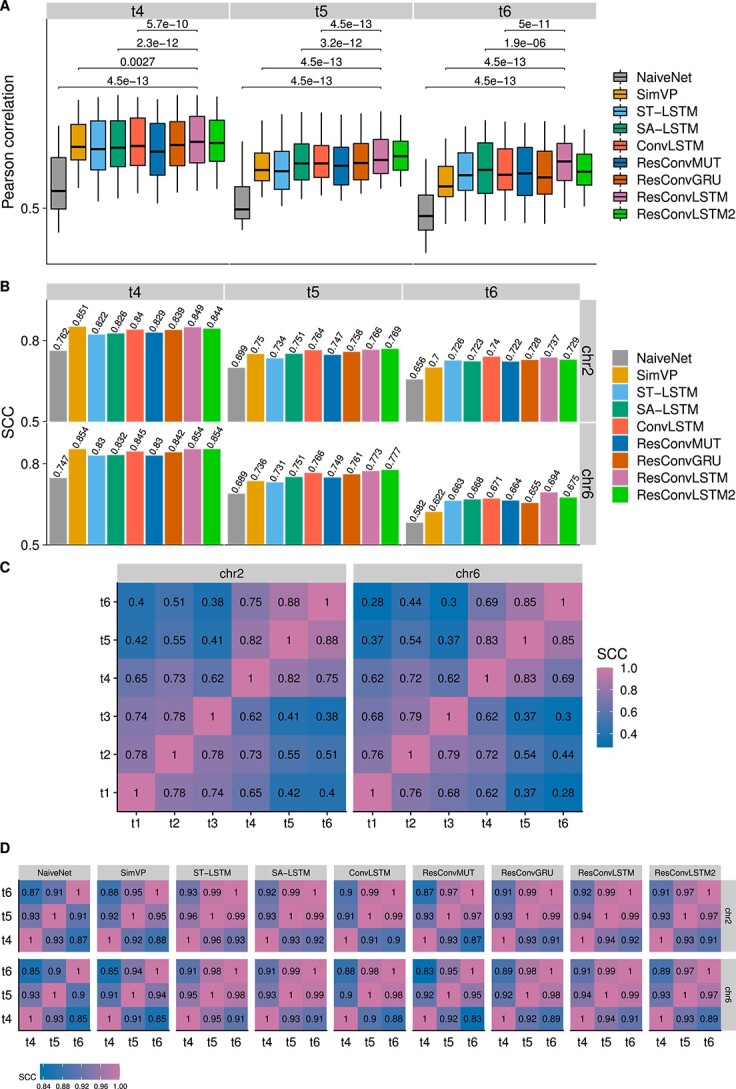
Reproducibility results on dataset 1. (**A**) Boxplots of Pearson correlations between ground truth and predicted Hi-C contact matrices at each genomic distance by pooling data from the two testing chromosomes (2 and 6). For clarity, only the P-values between ResConvLSTM and each of the other four methods (NaiveNet, SimVP, SA-LSTM and ConvLSTM) were shown. P-values were computed using the paired Wilcoxon test. (**B**) SCC scores between ground truth and predicted Hi-C contact matrices from the nine methods. (**C**) SCC scores between ground-truth Hi-C matrices from each pair of the six time-steps for two testing chromosomes. (**D**) SCC scores between predicted Hi-C matrices from each pair of the three predicted time-steps.

The reproducibility results for the blind test on dataset 1 are shown in Figure 2. Our newly designed ResConvLSTM often had significantly higher Pearson correlations in comparison with the other methods. The performance of SimVP became worse at the long-term time point ($t_{6}$). Moreover, ResConvLSTM mostly outperformed the other methods when the SCC scores for reproducibility were assessed. One exception is that SimVP achieved a bit higher SCC of 0.851 on chromosome 2 at time $t_{4}$ compared with 0.849 of ResConvLSTM. As expected, NaiveNet consistently had the lowest correlations and SCCs. Based on the sum of all SCC scores, the top three methods were ResConvLSTM, ResConvLSTM2 and ConvLSTM (Table S10). Next, we checked the similarity levels of Hi-C contact matrices between different time-steps. For ground-truth Hi-C, we calculated SCC scores between each pair of all six time-steps (Figure 2C). The small values in the upper left and lower right regions indicate that the three input time-steps are different from the three future time-steps. We also calculated SCC scores between each pair of the three predicted time-steps (Figure 2D), suggesting that the two three-step-ahead methods can forecast more distinct matrices than the other next-frame methods.

**Figure 3:**
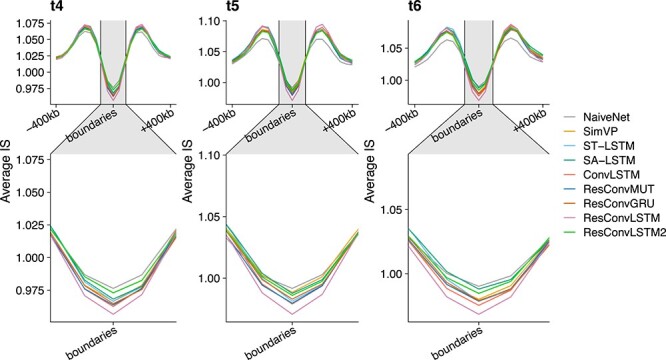
TAD-recovering results on dataset 1. Valleys/minima of insulation scores from predicted Hi-C contact matrices around strong TAD boundaries that were called based on ground-truth Hi-C.

**Figure 4:**
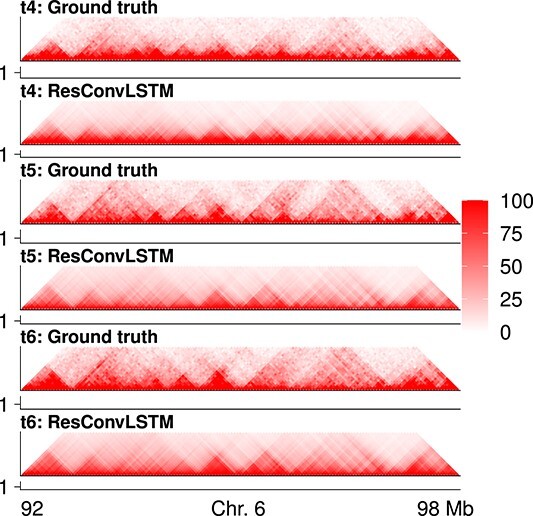
TAD-recovering results on chromosome 6 for dataset 1. Hi-C heat maps and their insulation-score curves for ground truth and ResConvLSTM predictions at the three future time-steps ($t_{4}$, $t_{5}$ and $t_{6}$).

The TAD-recovering results are shown in Figures 3–4 and Figure S6. We first found that almost all predicted Hi-C contact matrices had very similar insulation scores with ground truth (Figure S6), and even NaiveNet achieved >0.86 correlations at times $t_{4}$ and $t_{5}$. We then called strong TAD boundaries on ground-truth Hi-C contact matrices using cooltools (https://github.com/open2c/cooltools), plotted average insulation scores around strong TAD boundaries (Figure 3), and observed valleys/minima of average insulation scores calculated on the predicted Hi-C contact matrices from each of the five methods, especially for ResConvLSTM, indicating that these methods can successfully recover the strong TAD boundaries. Moreover, we showed some specific TAD-recovering examples at a genomic region in Figure 4 and Figure S7, revealing that our newly designed ResConvLSTM and the other methods can successfully recover TAD patterns that have not yet been fully established at the input time-steps.

In addition, we tested the effects of different numbers of input time-steps. Three more numbers of input time-steps (1, 2 and 4) are investigated, and the corresponding numbers of predicted time-steps are 5, 4 and 2, respectively. Four methods were retrained based on different numbers of input time-steps (details see supplementary materials), including NaiveNet, SimVP, ConvLSTM and ResConvLSTM. The evaluation results are shown in Figure S8 for chromosome 2 and Figure S9 for chromosome 6, indicating that for all of the four methods, more time-steps used for training results in better performance. We suggest using the first three time-steps as input if the SCC score of 0.7 is an acceptable accuracy threshold for all predicted Hi-C matrices. These results also indicate that if the SCC of 0.6 is acceptable, the upper limit of the number of the predicted time-steps can reach five when using one time-step as the input.

### Benchmarks on datasets 2 and 3

**Figure 5:**
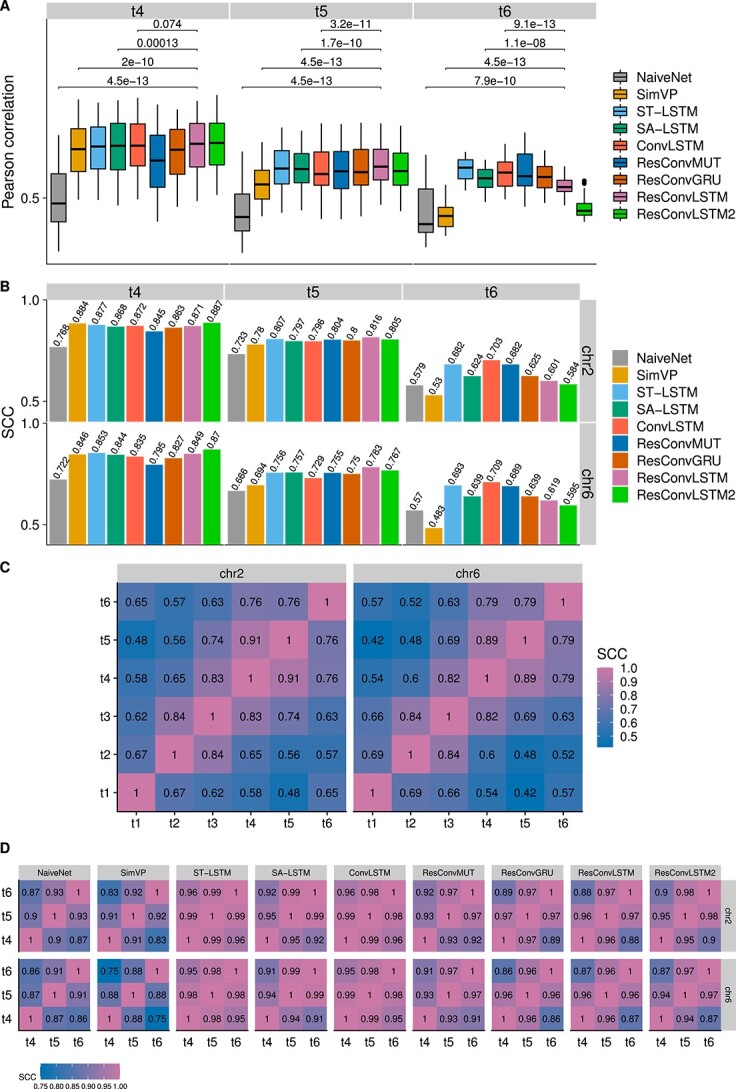
TAD-recovering results for dataset 2. (**A**) Boxplots of Pearson correlations for the two testing chromosomes (2 and 6). P-values were computed using the paired Wilcoxon test. (**B**) SCC scores between ground truth and predicted Hi-C contact matrices from the nine methods. (**C**) SCC scores between ground-truth Hi-C matrices from each pair of the six time-steps for two testing chromosomes. (**D**) SCC scores between predicted Hi-C matrices from each pair of the three predicted time-steps.

It is worth noting that compared with the other datasets dataset 2 is more similar to dataset 1 because of the number of read pairs after downsampling, time-point ranking order and the fact that they are both from mouse embryogenesis. We report the reproducibility benchmarks in Figure 5A and B and TAD-recovering results in Figures S10–S12. At the first future time-step $t_{4}$, ResConvLSTM2 often had the highest correlations and SCCs. At time $t_{5}$, ResConvLSTM significantly outperforms the others. However, ConvLSTM performed best at time $t_{6}$ and was closely followed by ST-LSTM and ResConvMUT. SimVP somehow performed worse even than NaiveNet at time $t_{6}$. The top three methods based on the sum of all SCC scores are ST-LSTM, ConvLSTM and ResConvMUT (Table S10). As we concluded for dataset 1, the TAD-recovering results (Figures S10–S12) for dataset 2 also indicated that the five methods can successfully recover TAD profiles even though our models were not trained on this dataset. Moreover, we generated SCC heat maps for ground truth (Figure 5C) and predicted Hi-C matrices (Figure 5D), suggesting that the first three time-steps are also markedly different from the last three and that next-frame methods are prone to generating a matrix that looks more like its previous prediction than the three-step ahead methods.

The third dataset contains 13 time-points, and we only used six of them for matching the time-steps of dataset 1. The main difference between dataset 3 and the first two datasets is that spatiotemporal Hi-C data were captured during SCNT embryo development in dataset 3, whereas it was mouse embryos for the first two datasets. The reproducibility benchmarks for dataset 3 are shown in Figure S13. In general, SA-LSTM and ResConvLSTM perform slightly better than the other methods, and the two three-step ahead methods perform noticeably worse than the next-frame methods. The three methods (SA-LSTM, ResConvLSTM and ResConvLSTM2) achieved the top three SCC scores (Table S10). The SCC heat maps (Figure S13C and S13D) are very similar to what we observed from the first two datasets.

### Performances on embryogenesis datasets from different species

**Figure 6:**
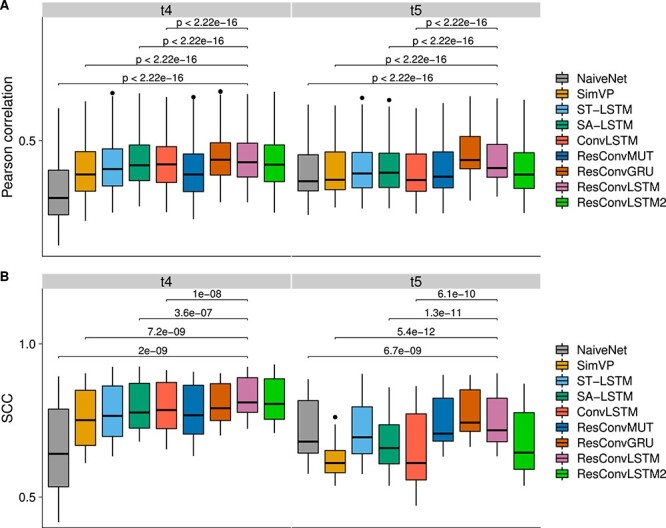
Reproducibility results for dataset 4. (**A**) Boxplots of Pearson correlations at each genomic distance. P-values were computed using the paired Wilcoxon test. (**B**) SCC scores between ground truth and predicted Hi-C contact matrices. P-values were computed using the paired t-test. Both correlation and SCC values were collected from all chromosomes.

The spatiotemporal Hi-C data in the fourth dataset were captured during human embryo development, which help us assess our methods in a different species. The reproducibility results for two future time-steps ($t_{4}$ and $t_{5}$) are shown in Figure 6. At time $t_{4}$, the top three methods are the residual networks (ResConvLSTM, ResConvLSTM2 and ResConvGRU). At time $t_{5}$, ResConvGRU performs best followed by ResConvLSTM, whereas ConvLSTM and SimVP perform worse even than NaiveNet. The top three methods that achieve the highest sum of all SCCs are the three residual networks (Table S10), including ResConvGRU, ResConvLSTM and ResConvMUT. As we observed from the evaluation results for the first three datasets, it seems that SimVP cannot predict long-term Hi-C matrices as well as predicting short-term ones, whereas our method ResConvLSTM does not have this weakness. The SCC heat map for the ground-truth Hi-C is shown in Figure S14, indicating that the Hi-C matrices for each time-step are distinct from each other. In addition, we reported the running time and memory consumption for predicting dataset 4 in Figure S15. Residual networks usually took more time to make predictions, but still no more than five minutes for predicting one chromosome.

**Figure 7:**
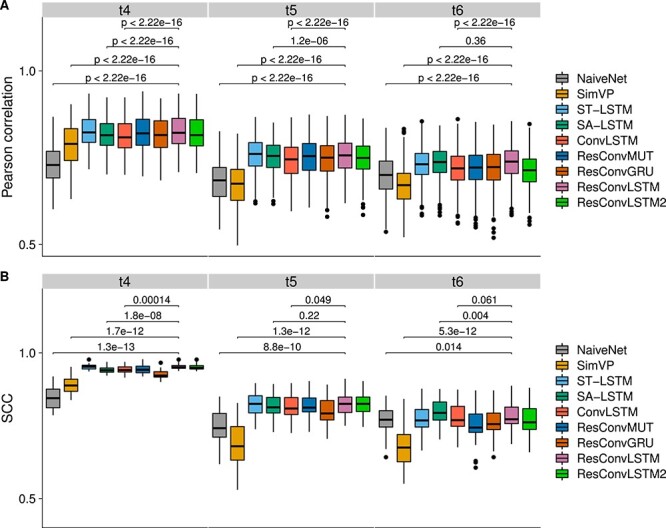
Reproducibility results for dataset 5. (**A**) Boxplots of Pearson correlations at each genomic distance. P-values were computed using the paired Wilcoxon test. (**B**) SCC scores between ground truth and predicted Hi-C contact matrices. P-values were computed using the paired t-test.

Datasets 5 and 6 are from two different species (medaka and X. tropicalis). The SCC heat maps (Figure S16 for dataset 5 and Figure S17 for dataset 6) indicate that the three input time-steps are distinct from the last three time-steps, especially for dataset 6. The reproducibility results are shown in Figure 7 for dataset 5 and Figure S18 for dataset 6. For dataset 5, ResConvLSTM performs best at times $t_{4}$ and $t_{5}$, while at time $t_{6}$ SA-LSTM outperforms the others. The top three methods based on the sum of all SCC values are ResConvLSTM, SA-LSTM and ST-LSTM (Table S10). For dataset 6, the best three with the highest sum of SCC scores are ResConvLSTM, ResConvLSTM2,and ConvLSTM (Table S10).

### Performances on non-embryogenesis datasets

**Figure 8:**
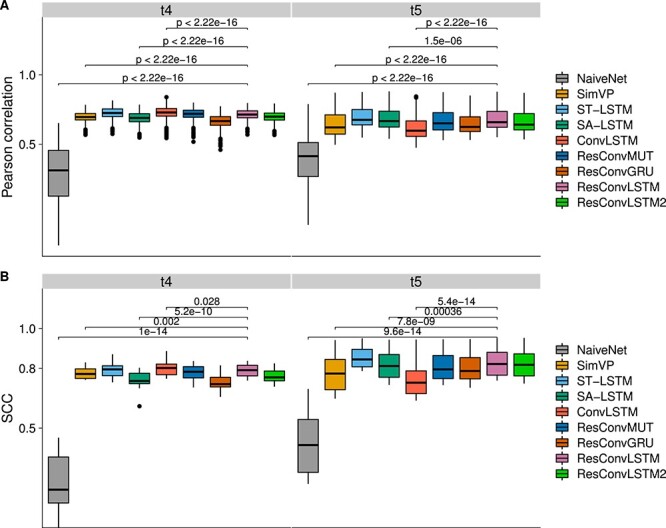
Reproducibility benchmarks for dataset 7. (**A**) Boxplots of Pearson correlations at each genomic distance. P-values were computed using the paired Wilcoxon test. (**B**) SCC scores between ground truth and predicted Hi-C contact matrices. P-values were computed using the paired t-test.

The last two datasets (7 and 8) were selected to benchmark the nine methods on non-embryogenesis data. Since the spatiotemporal Hi-C from datasets 7 and 8 are not related to embryogenesis, we used all chromosomes for blind testing. The SCC heat maps are shown in Figure S19 for dataset 7 and Figure S20 for dataset 8, which indicate that the spatiotemporal Hi-C data from non-embryogenesis developments are more indistinguishable than those from embryogenesis. The evaluation results for the two datasets are shown in Figure 8 and Figure S21, respectively. The top two methods for both datasets are the same (ST-LSTM and ResConvLSTM) and the third method is ResConvMUT for dataset 7 and ResConvLSTM2 for dataset 8 (Table S10), suggesting that when predicting non-embryogenesis spatiotemporal Hi-C ST-LSTM and residual networks may be better choices.

## DISCUSSION AND CONCLUSION

In this paper, we present HiC4D for exploring the forecasting problem of spatiotemporal Hi-C data. We predicted the future three Hi-C frames from the previous three frames with nine different video-prediction methods. Specifically, we reimplemented three RNN methods (ConvLSTM, ST-LSTM and SA-LSTM), introduced a novel method (ResConvLSTM) together with its three variants (ResConvGRU, ResConvMUT and ResConvLSTM2) and used a state-of-the-art method (SimVP) and a NaiveNet as a baseline. These methods were trained with the same data and blindly tested on eight different spatiotemporal Hi-C datasets. Our benchmarks indicate that our newly designed method ResConvLSTM or its variants almost always outperforms the other methods across the eight datasets by achieving higher reproducibility scores. Our evaluation results also indicate that all methods can successfully recover TAD boundaries. Moreover, we show that models learned from one species can be used to forecasting the spatiotemporal Hi-C of another species. Together, HiC4D is an effective tool for accurately predicting spatiotemporal Hi-C data.

**Key Points**

- A novel topic of forecasting spatiotemporal Hi-C with deep learning methods is introduced. To the best of our knowledge, this work is the first computational method to forecast spatiotemoral Hi-C data with deep learning methods.
- We present HiC4D, a newly designed method for accurately forecasting spatiotemporal Hi-C data. The method combines residual networks and convolutional long short-term memory (ConvLSTM) that makes ConvLSTM “deeper” (having more layers) and better learn the dependencies from the data.
- We benchmarked nine deep networks. Our evaluation results indicate that residual RNN networks almost always outperform the other methods on eight different spatiotemporal Hi-C datasets in terms of recovering Hi-C contacts and boundaries of TADs.

## Supplementary Material

HiC4D_suppl_2_bbad263

## Data Availability

The source code of HiC4D can be found at both http://dna.cs.miami.edu/HiC4D/ and https://github.com/zwang-bioinformatics/HiC4D/. The data sources used in this paper are reported in the section “MATERIALS AND METHODS”. All of the datasets generated in this study are available upon request.

## Author Biographies

**Tong Liu** is an Assistant Scientist in the Department of Computer Science at the University of Miami.

**Zheng Wang** is an Associate Professor in the Department of Computer Science, Department of Biology and Sylvester Comprehensive Cancer Center at the University of Miami.
