# Supplementary material for: HiC4D: forecasting spatiotemporal Hi-C data with residual ConvLSTM
Source: Brief Bioinform. 2023 Jul 20;24(5):bbad263. doi: 10.1093/bib/bbad263 (PMC10516390; doi:10.1093/bib/bbad263)
Supplement: HiC4D_suppl_2_bbad263 [file hic4d_suppl_2_bbad263.pdf]

HiC4D: Forecasting spatiotemporal Hi-C data with residual ConvLSTM

Supplementary Materials

ConvLSTM-2

$$i_t = \sigma(W_{xi} * X_t + W_{hi} * H_{t-1}^l + b_i)$$
$$f_t = \sigma\left(W_{xf} * X_t + W_{hf} * H_{t-1}^l + b_f\right)$$
$$C_t^l = f_t \circ C_{t-1}^l + i_t \circ \tanh(W_{xc} * X_t + W_{hc} * H_{t-1}^l + b_c)$$
$$o_t = \sigma(W_{xo} * X_t + W_{ho} * H_{t-1}^l + b_o)$$
$$H_t^l = o_t \circ \tanh\,(C_t^l)$$

ConvLSTM-3

$$i_t = \sigma(W_{xi} * X_t + W_{hi} * H_{t-1}^l + W_{ci} * C_{t-1}^l + b_i)$$
$$f_t = \sigma\left(W_{xf} * X_t + W_{hf} * H_{t-1}^l + W_{cf} * C_{t-1}^l + b_f\right)$$
$$C_t^l = f_t \circ C_{t-1}^l + i_t \circ \tanh(W_{xc} * X_t + W_{hc} * H_{t-1}^l + b_c)$$
$$o_t = \sigma(W_{xo} * X_t + W_{ho} * H_{t-1}^l + W_{co} * C_t^l + b_o)$$
$$H_t^l = o_t \circ \tanh\,(C_t^l)$$

Gated Recurrent Unit (GRU)

$$z_t = \sigma(W_{xz} * X_t + W_{hz} * H_{t-1}^l + b_z)$$
$$r_t = \sigma(W_{xr} * X_t + W_{hr} * H_{t-1}^l + b_r)$$
$$\widetilde{H}_t^l = \tanh(W_{xh} * X_t + W_{hh} \circ H_{t-1}^l + b_h)$$
$$H_t^l = z_t \circ H_{t-1}^l \; + \; (1-z_t) \circ \widetilde{H}_t^l$$

MUT

$$z_t = \sigma(W_{xz} * X_t + b_z)$$
$$r_t = \sigma(W_{xr} * X_t + W_{hr} * H_{t-1}^l + b_r)$$
$$H_t^l = z_t \circ \tanh(W_{hh} * (r_t \circ H_{t-1}^l) + tanh(X_t) + b_h) \; + \; (1-z_t) \circ \; H_{t-1}^l$$

Insulation score

The insulation score (IS) for the i-th 40-kb bin is calculated as following:

$$IS_i = \frac{1}{n^2} \sum_{j \in J, k \in K} C(j, k)$$

$$IS'_i = \log_2(\frac{IS_i}{IS_{avg}} + 1),$$

where J and K are the bin sets of $[i - 5, i - 1]$ and $[i + 1, i + 5]$, respectively, $C(j, k)$ is the Hi-C contacts at bin indexes j and k, n is equal to 5, and IS_{avg} is the average IS of all bins on the current chromosome. The final IS (IS'_i) is the log transformation of $\log_2(x + 1)$, where x is the ratio of each bin's IS (IS_i) and the average IS (IS_{avg}).

Different lengths of input time steps

We used the four methods (two next-frame methods ConvLSTM and ResConvLSTM, two n-step ahead methods NaiveNet and SimVP) for testing different lengths of input time steps. It was easier for the two next-frame methods to change the lengths of input time steps by resetting from which time step we start to use the reconstructed matrix as the input of next time. For the two n-step ahead methods, we usually need to revise the network by appending an output layer or resetting the last out channels to obtain predicted matrices with the desired lengths of predicted time steps. For example, if we want to use NaiveNet to predict five time-steps with only one time step as input ($1 \rightarrow 5$), we just set the output channel of the last layer to five.

Table S1. Details of dataset 1 about numbers of valid read pairs at each time step.

Time ID	Time points	Number of allValidPairs	After filtering (>20kb intra-chr.)	After downsampling
t1	PN5 zygote	250,033,607	136,514,531	115,000,000
t2	Early 2-cell	238,136,944	134,350,801	
t3	Late 2-cell	309,906,331	170,077,578	
t4	8-cell	290,254,185	192,683,578	
t5	ICM	221,486,743	149,431,330	
t6	mESC	158,789,957	115,438,254	

Table S2. Details of dataset 2 about numbers of contact pairs at each time step.

Time ID	Time points	Number of all contact pairs	After filtering (>20kb intra-chr.)	After downsampling
t1	zygote	254,205,558	62,176,913	62,000,000
t2	2-cell	234,656,744	64,917,644	
t3	4-cell	656,846,297	209,313,083	
t4	8-cell	572,517,384	153,136,307	
t5	E3.5	596,167,520	178,305,007	
t6	E7.5	1,412,804,902	575,229,812	

Table S3. Details of dataset 3 about numbers of valid read pairs at each time step.

Time ID	Time points	Number of allValidPairs	After filtering (>20kb intra-chr.)	After downsampling
	CC	137,231,961	96,131,399	33,000,000
	0.5hpi	13,829,278	9,385,399	
	1hpi	11,321,468	8,590,129	
	1hpa	8,926,567	7,148,171	
	6hpa	113,494,879	56,164,146	
t1	12hpa	159,766,544	78,174,527	
t2	Early-2-cell	174,948,961	94,369,270	
t3	Late-2-cell	89,978,789	53,398,992	
	4-cell	100,220,085	54,414,433	
t4	8-cell	51,205,945	33,723,297	
	Morula	87,366,689	57,708,483	
t5	ICM	123,165,649	80,643,557	
t6	TE	165,825,942	113,567,317	

Table S4. Details of dataset 4 about numbers of contact pairs at each time step.

Time ID	Time points	Number of all contact pairs	After filtering (>20kb intra-chr.)	After downsampling
t1	2-cell	40,311,069	14,733,663	14,500,000
t2	8-cell	69,829,968	25,458,239	
t3	morula	43,918,745	14,557,114	
t4	blastocyst	755,470,274	310,951,826	
t5	6-week	596,229,576	235,384,135	

Table S5. Details of dataset 5 for medaka.

Time ID	Time points	Number of all contact pairs	After filtering (>20kb intra-chr.)	After downsampling
t1	ST11	321,990,213	75,599,447	56,000,000
t2	ST12	287,602,004	72,177,281	
t3	ST13	302,812,966	56,793,710	
t4	ST14	330,920,449	64,797,800	
t5	ST18	330,008,317	75,943,643	
t6	ST27	314,273,388	71,377,630	

Table S6. Details of dataset 6 for Xenopus tropicalis.

Time ID	Time points	Number of all contact pairs	After filtering (>20kb intra-chr.)	After downsampling
t1	ST8	324,299,078	83,144,547	50,000,000
t2	ST9	427,288,415	168,181,258	
t3	ST10	364,737,811	136,768,063	
t4	ST12	399,598,123	158,280,826	
t5	ST15	339,656,948	127,200,374	
t6	ST23	333,589,316	116,940,684	

Table S7. Details of dataset 7 for human cardiogenesis.

Time ID	Time points	Number of all contact pairs	After filtering (>20kb intra-chr.)	After downsampling
t1	hESC	138,646,497	76,487,810	56,000,000
t2	MES	143,513,886	60,357,784	
t3	CP	161,879,671	69,214,205	
t4	CM	185,518,894	56,446,512	
t5	Fetal heart	153,137,875	72,582,488	

Table S8. Details of dataset 8 for mouse cell reprogramming.

Time ID	Time points	Number of all contact pairs	After filtering (>20kb intra-chr.)	After downsampling
t1	B	2,361,772,475	844,361,188	150,000,000
t2	Ba	2,219,612,821	820,482,947	
t3	D2	2,343,041,485	882,828,358	
t4	D4	2,207,227,520	771,632,880	
t5	D6	2,260,473,500	852,796,687	
t6	D8	2,420,686,998	786,512,233	

Table S9. Validation results of hyperparameter tuning for three next-frame methods (ConvLSTM, ResConvLSTM, and ST-LSTM) and two 3-step ahead methods (SimVP and NaiveNet). The further evaluation results for the highlighted models are shown in Results section. Three ResConvLSTM variants (ResConvGRU, ResConvMUT, and ResConvLSTM2) are trained with hyperparameters same as the final ResCOnvLSTM we used. The hidden dimensions in parentheses for SA-LSTM are for self-attention module.

Method	Loss	Batch size	Hidden dimension	Kernel size	Number of layers	Validation loss
ConvLSTM-1	MSE	32	128	5	4	0.00676
		16	128	5	8	0.00694
		16	128	5	12	0.00686
		**32**	**128**	**7**	**4**	**0.00670**
		32	128	11	4	0.00677
		32	128	7	2	0.00673
		32	32	7	4	0.00679
ConvLSTM-2	MSE	32	128	5	4	0.00672
		**32**	**128**	**7**	**4**	**0.00669**
		32	128	7	2	0.00678
		32	128	9	4	0.00671
ConvLSTM-3	MSE	32	128	5	4	0.00684
		**32**	**128**	**7**	**4**	**0.00687**
ResConvLSTM	MSE	32	128	7	6	0.00669
		**32**	**128**	**7**	**10**	**0.00666**
		32	128	7	14	0.00676
		**32**	**64**	**7**	**30**	**0.00664**
		32	64	5	30	0.00677
		**32**	**32**	**7**	**52**	**0.00666**
		32	16	7	102	0.00673
ST-LSTM	MSE	**32**	128	**5**	**4**	**0.00667**
		16	128	5	8	0.00683
		16	128	5	12	0.00682
		32	128	7	4	0.00676
		32	128	11	4	0.00675
	MSE + decouple	**32**	**128**	**5**	**4**	**0.00690**
		**32**	**128**	**7**	**4**	**0.00689**
ResConvGRU	MSE	32	32	7	52	0.00666
ResConvMUT						0.00675
ResConvLSTM2						0.00678
SA-LSTM	MSE	32	32(4)	7	4	0.00679
		16	64(4)	7	4	0.00682
		**16**	**128(4)**	**7**	**4**	**0.00672**
		16	128(32)	7	4	0.00675
SimVP	MSE	32	128	-	-	0.00944
NaiveNet	MSE	32	128	7	3	0.01105

Table S10. The top 3 methods that having the largest sum of all SCC scores for each of the eight datasets.

Data set cluster	Data set ID	Top 3 methods		
1	1	ResConvLSTM	ResConvLSTM2	ConvLSTM
	2	ST-LSTM	ConvLSTM	ResConvMUT
	3	SA-LSTM	ResConvLSTM	ResConvLSTM2
2	4	ResConvGRU	ResConvLSTM	ResConvMUT
	5	ResConvLSTM	SA-LSTM	ST-LSTM
	6	ResConvLSTM	ResConvLSTM2	ConvLSTM
3	7	ST-LSTM	ResConvLSTM	ResConvMUT
	8	ST-LSTM	ResConvLSTM	ResConvLSTM2

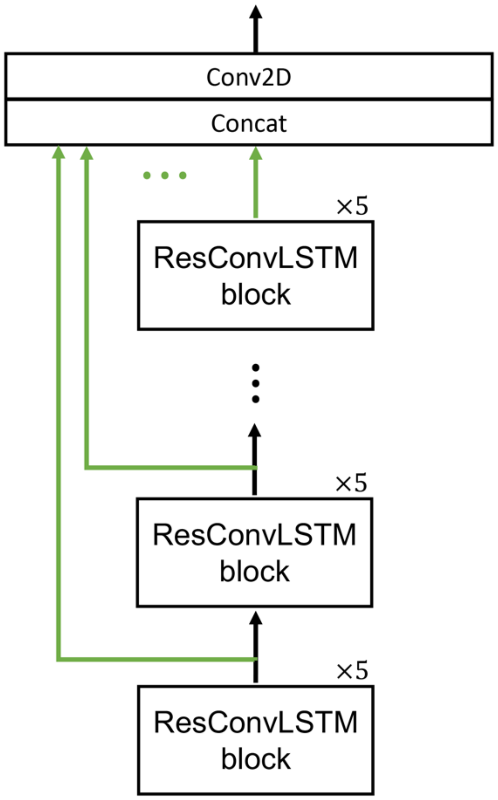

Fig. S1. The architecture of ResConvLSTM2. The outputs of every five ResConvLSTM blocks are concatenated as input for the final output layer.

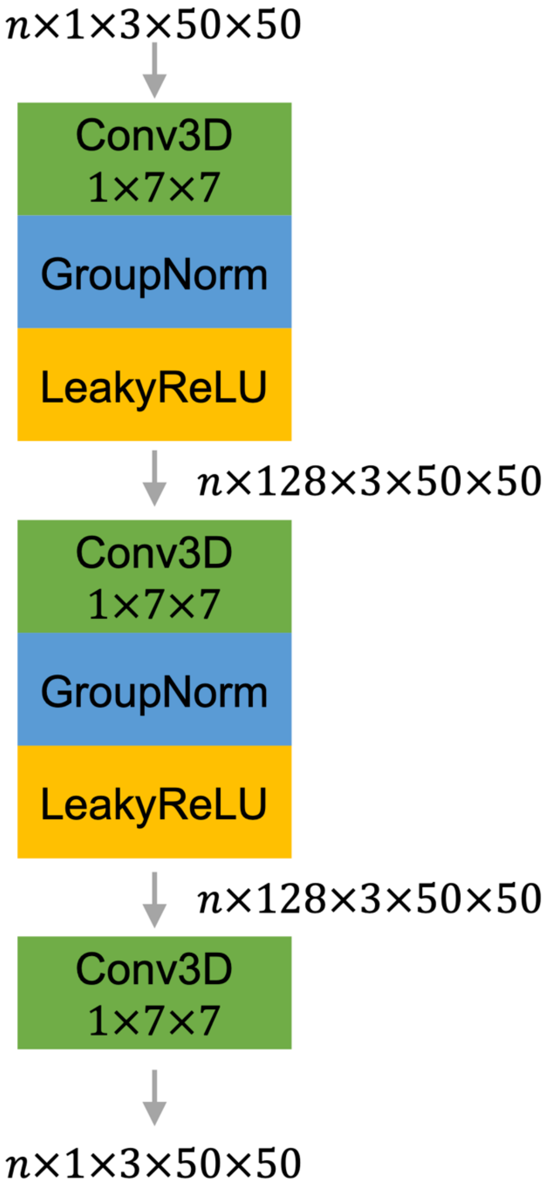

Fig. S2. The architecture of the 3-layer NaiveNet. The number of groups for each of the two group normalizations (GroupNorm) is set to 2. The negative slope for each of the two LeakyReLUs is set to 0.2. The padding tuple for each of the three Conv3Ds is (1, 3, 3) for keeping the shape of $3 \times 50 \times 50$. The three input temporal channels are t_1, t_2, and t_3, while the three output temporal channels are corresponding to t_4, t_5, and t_6.

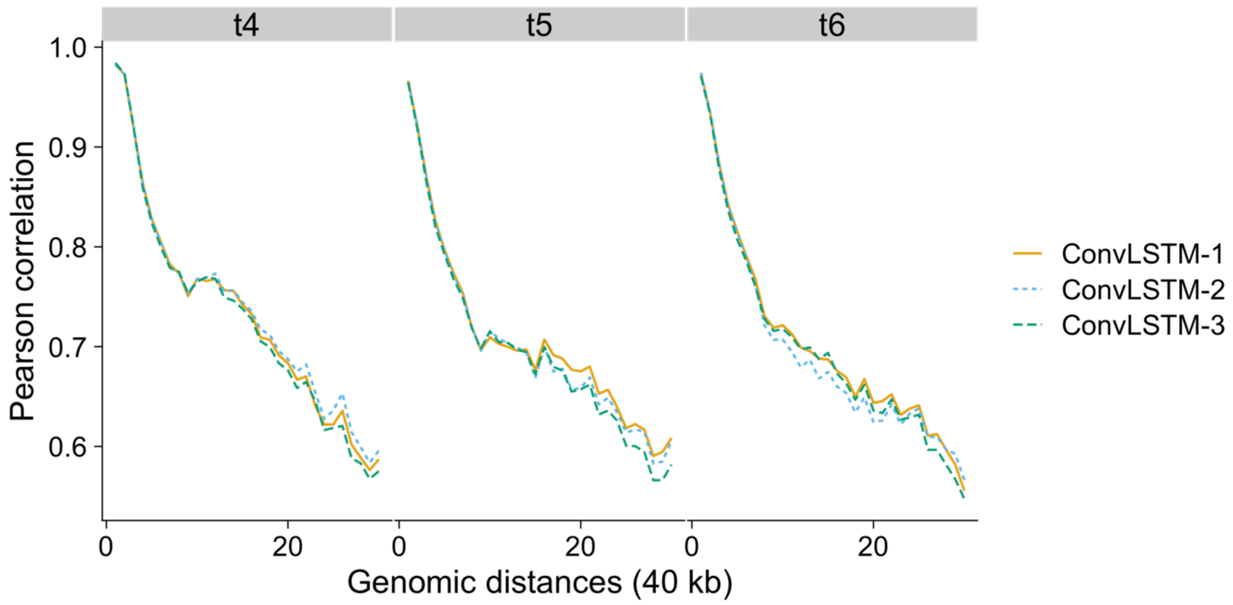

Fig. S3. Performances of three ConvLSTM networks on validation data from chromosome 19 at times t_4, t_5, and t_6. Pearson correlations between experimental Hi-C and predicted Hi-C from each of the three ConvLSTMs at each genomic distance. The three ConvLSTMs were trained with the same hyperparameters: the batch size of 32, the kernel size of 5, and the number of layers equal to 4.

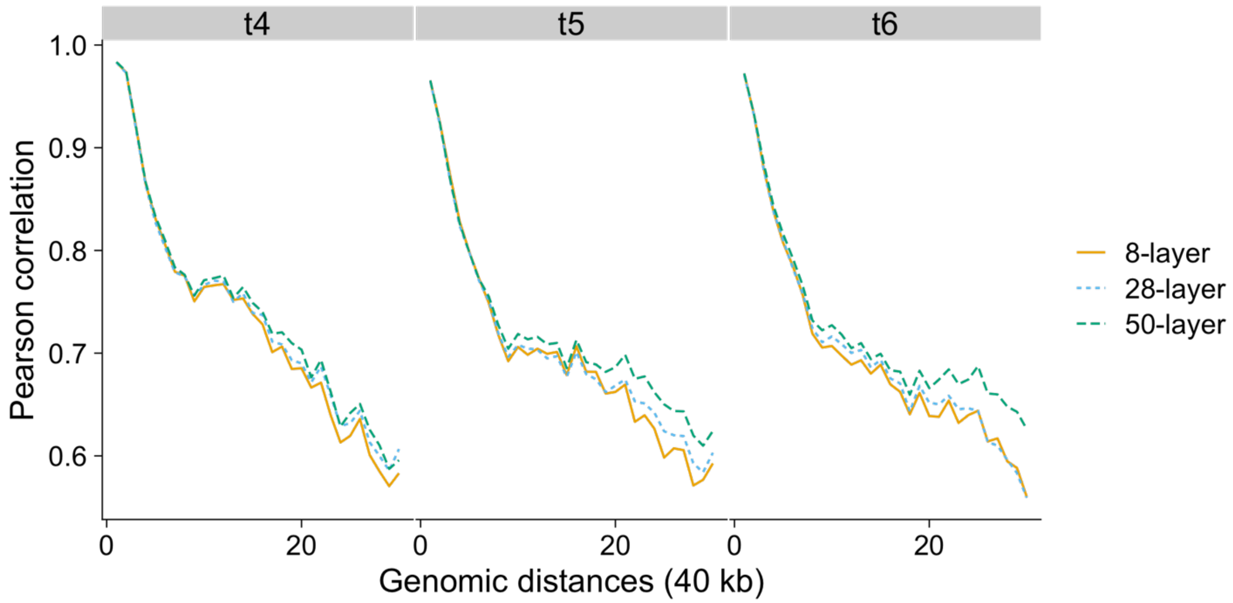

Fig. S4. Deeper ResConvLSTM performs better on validation data from chromosome 19. Pearson correlations between experimental Hi-C and predicted Hi-C from each of the three ResConvLSTMs at each genomic distance. The three ResConvLSTM networks were trained with the same kernel size of 7 and the same batch size of 32, but with different hidden dimensions and number of layers because of the limitation of GPU memory. The hidden dimensions of the 8-layer, 28-layer, and 50-layer networks (together with two more convolutional layers) are 128, 64, and 32, respectively.

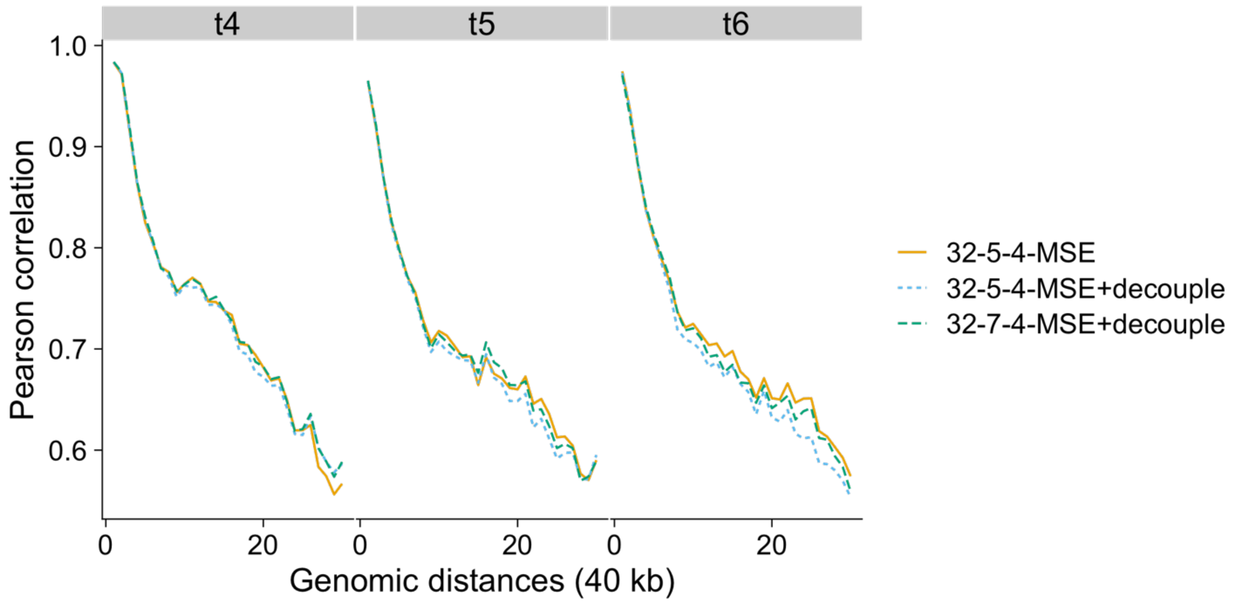

Fig. S5. ST-LSTM without decouple loss performs better on validation data from chromosome 19 at time t_6. Pearson correlations between experimental Hi-C and predicted Hi-C from each of the three ST-LSTMs at each genomic distance. The three ST-LSTMs were trained with different configurations. The first one was equipped with the batch size of 32, the kernel size of 5, and the number of layers equal to 4 together with only MSE loss. The second one was equipped with the same hyperparameters as the first one but used MSE plus decouple loss. The last one was equipped with the same batch size (32) and the number of layers (4), but with the kernel size of 7 together with MSE plus 0.1 times decouple loss.

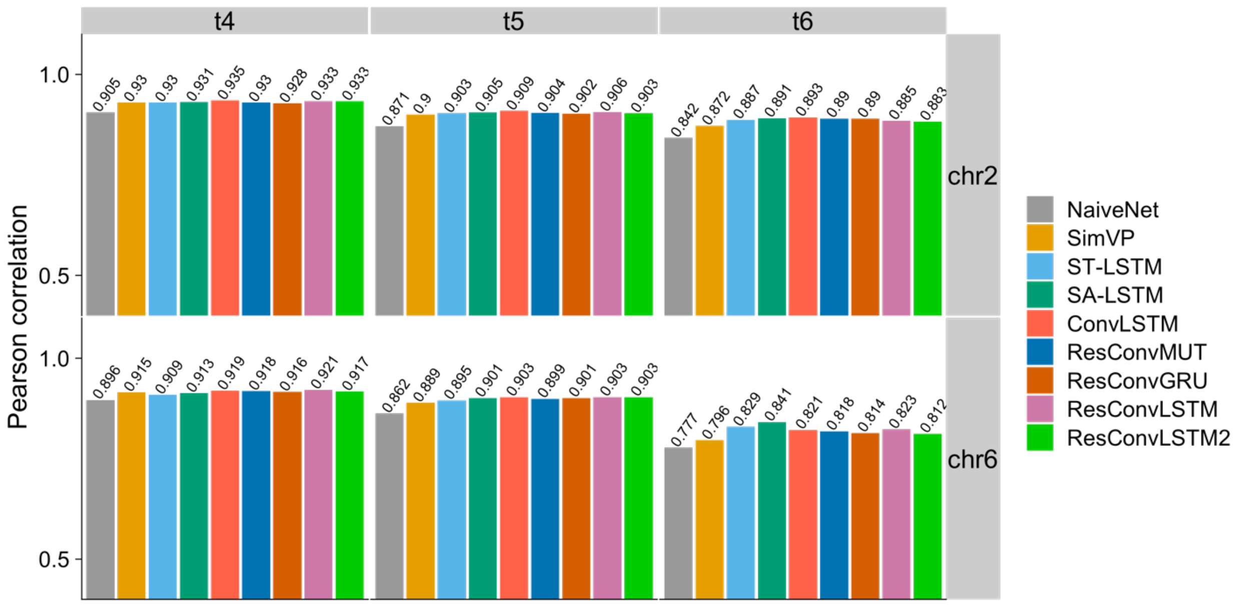

Fig. S6. Pearson correlations between insulation scores calculated on experimental (ground truth) and predicted Hi-C contact matrices from nine methods on dataset 1.

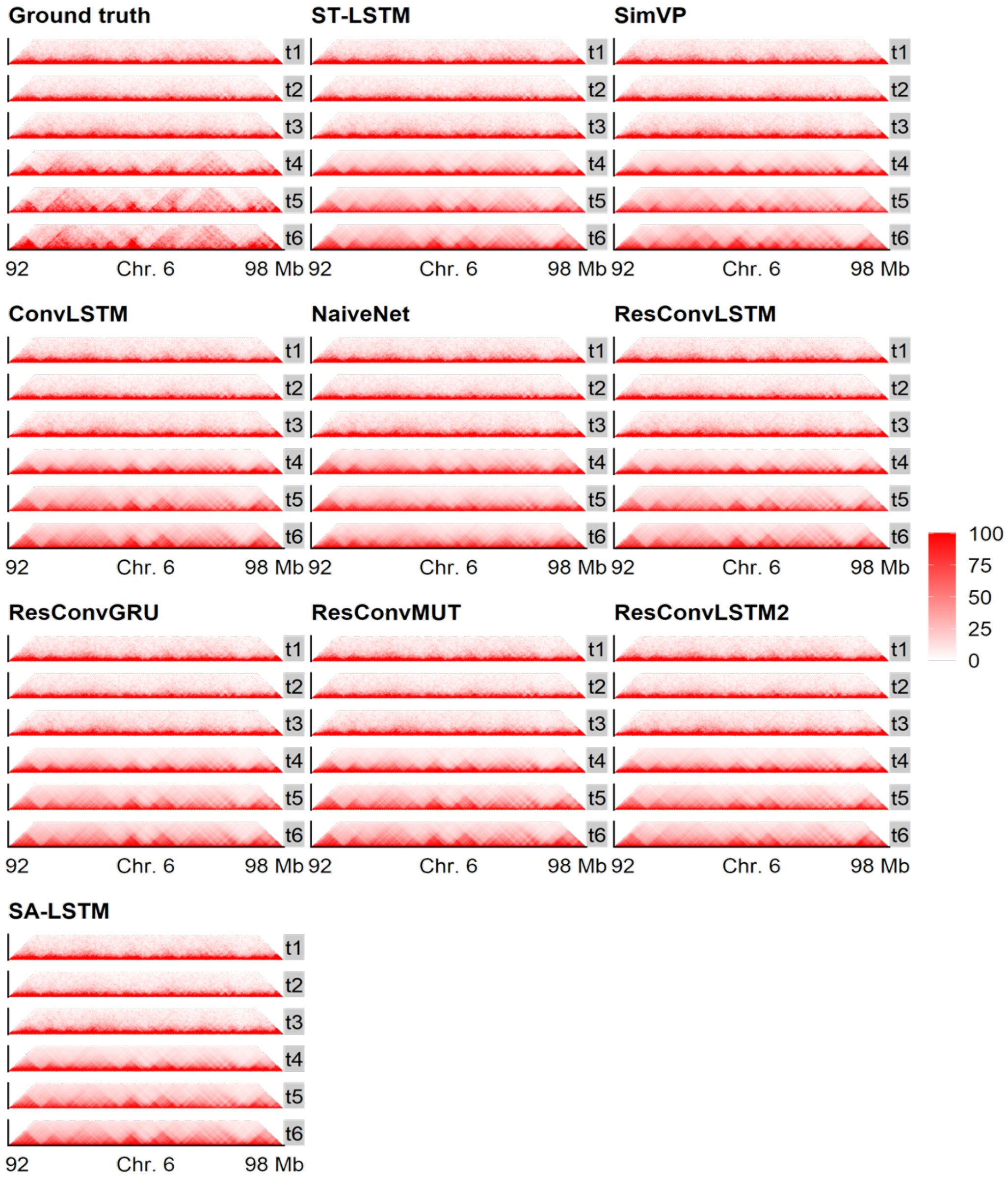

Fig. S7. Hi-C heat maps of ground truth and predictions from nine methods at the input (t_1, t_2, and t_3) and future (t_4, t_5, and t_6) time-steps on dataset 1.

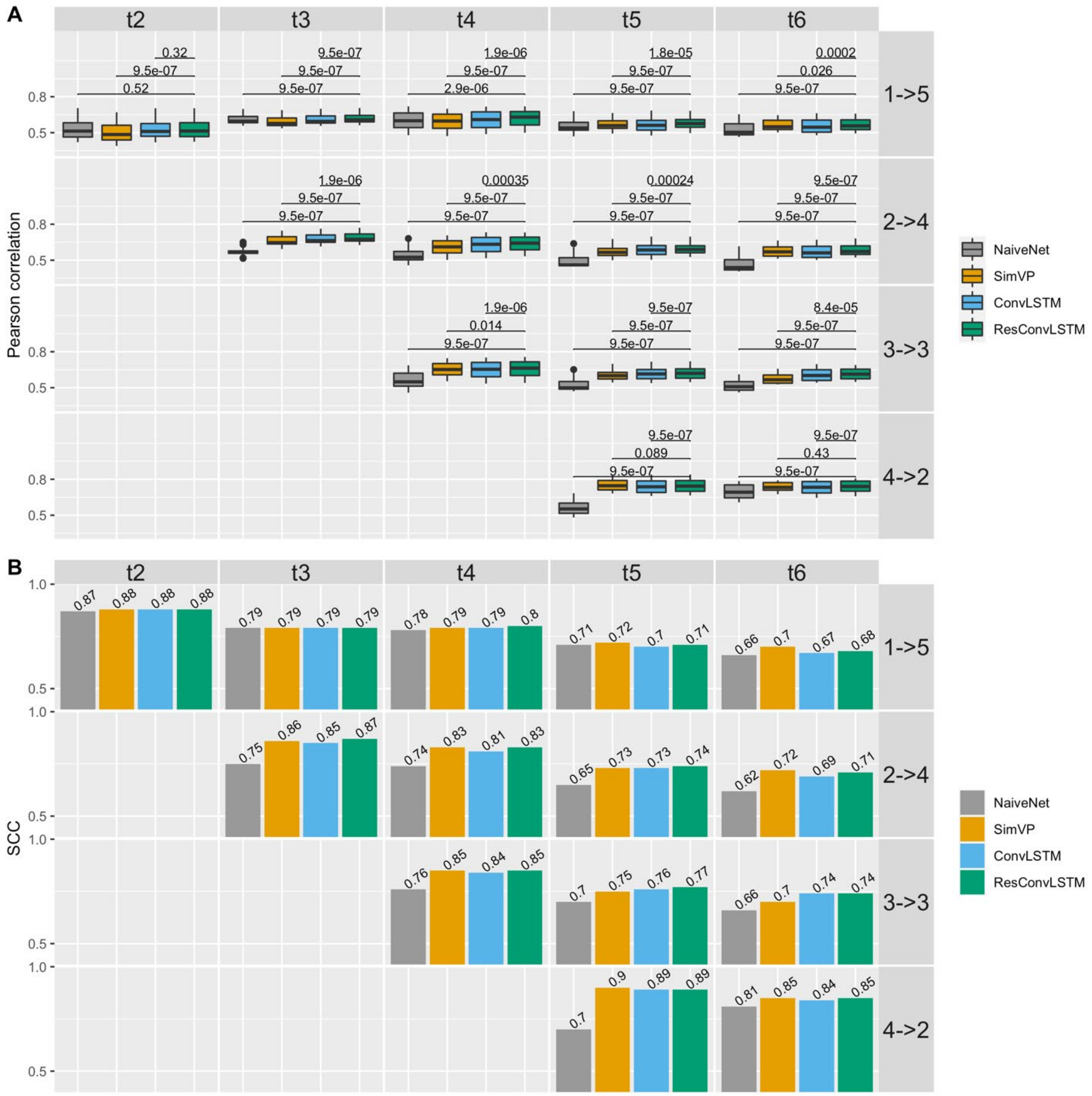

Fig. S8. Performances of four methods on chromosome 2 from dataset 1 for using different lengths of input time steps. (A) Boxplots of Pearson correlations between ground truth and predicted Hi-C contact matrices at each genomic distance. P-values were computed using the paired Wilcoxon test. (B) SCC scores between ground truth and predicted Hi-C contact matrices from the four methods.

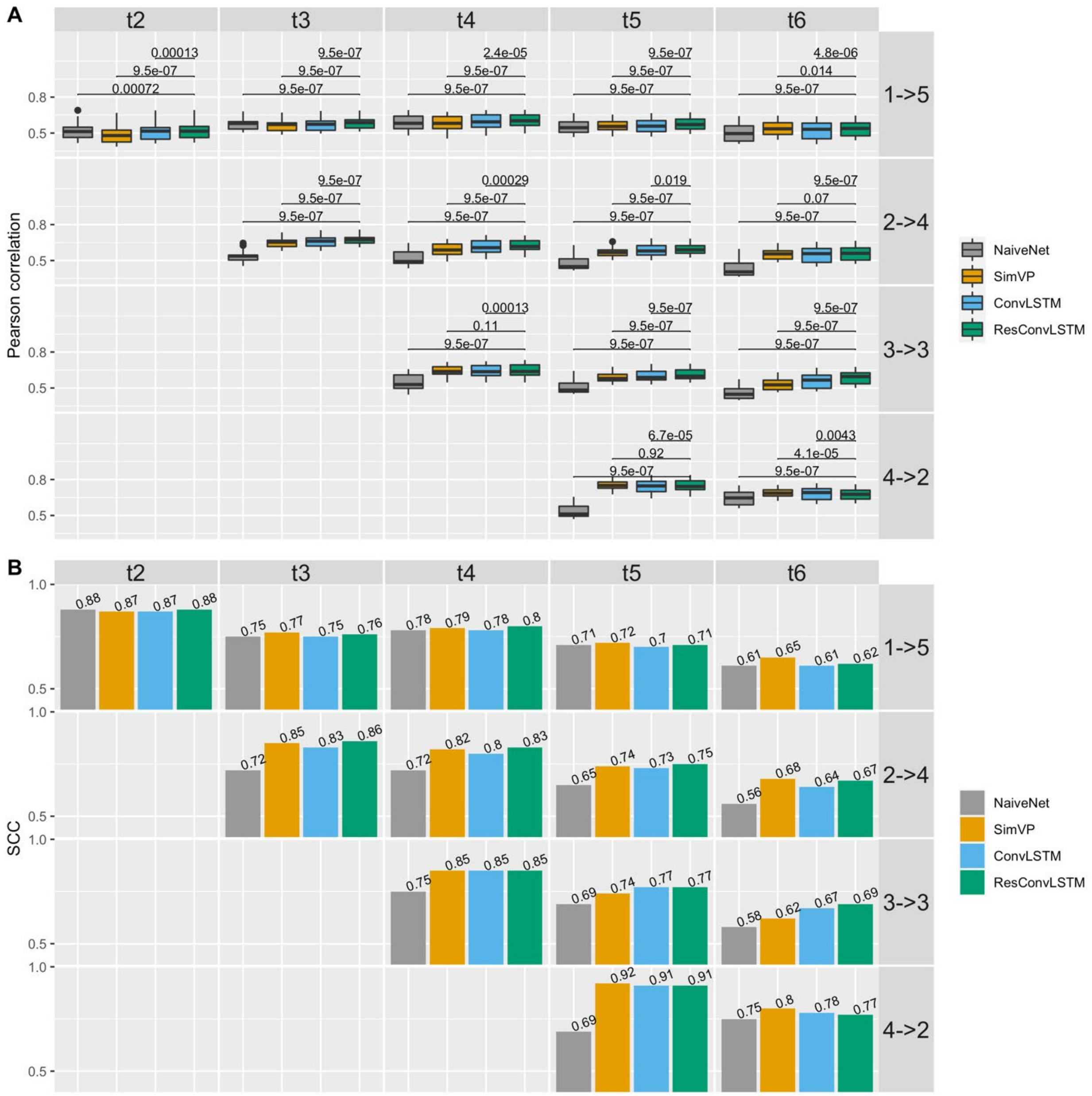

Fig. S9. Performances of the four methods on chromosome 6 from dataset 1 for using different lengths of input time steps. (A) Boxplots of Pearson correlations between ground truth and predicted Hi-C contact matrices at each genomic distance. P-values were computed using the paired Wilcoxon test. (B) SCC scores between ground truth and predicted Hi-C contact matrices from the four methods.

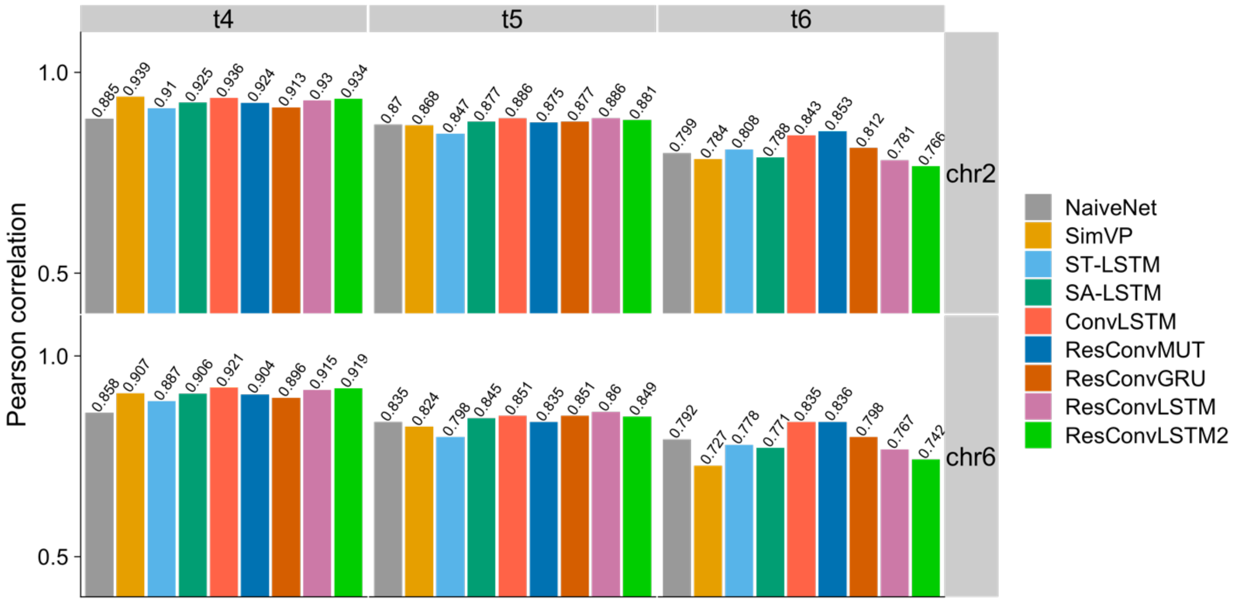

Fig. S10. Pearson correlations between insulation scores calculated on experimental (ground truth) and predicted Hi-C contact matrices on dataset 2.

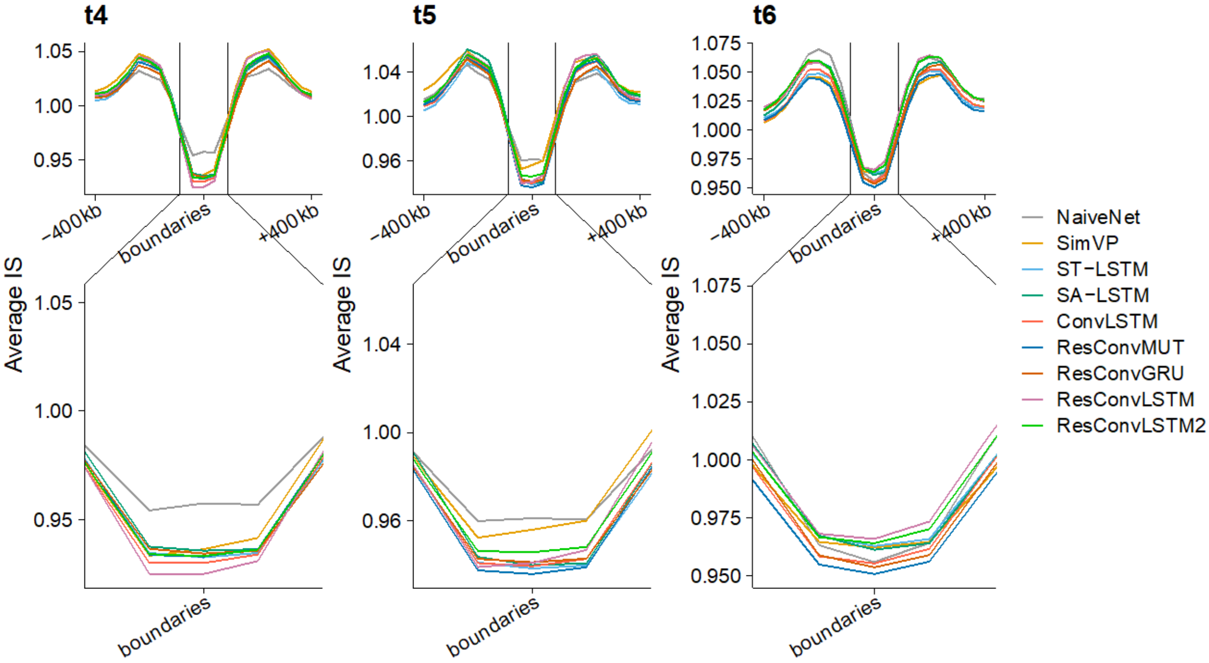

Fig. S11. TAD-recovering results on two testing chromosomes 2 and 6 for dataset 2. Valleys/minima of insulation scores from predicted Hi-C contact matrices around strong TAD boundaries called on experimental Hi-C.

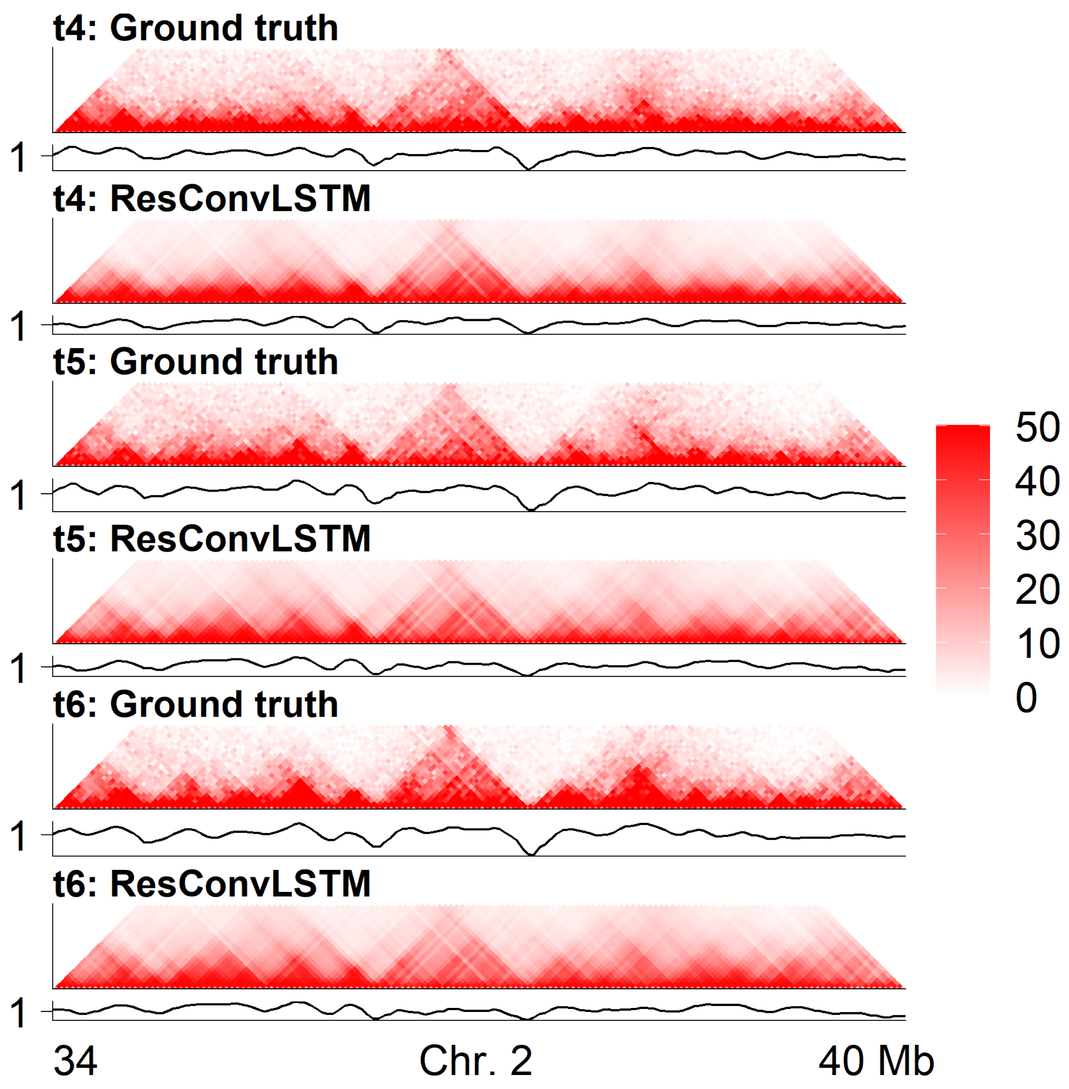

Fig. S12. TAD-recovering results on chromosome 2 for dataset 2. Hi-C heat maps and their corresponding insulation-score curves for ground truth and ResConvLSTM predictions.

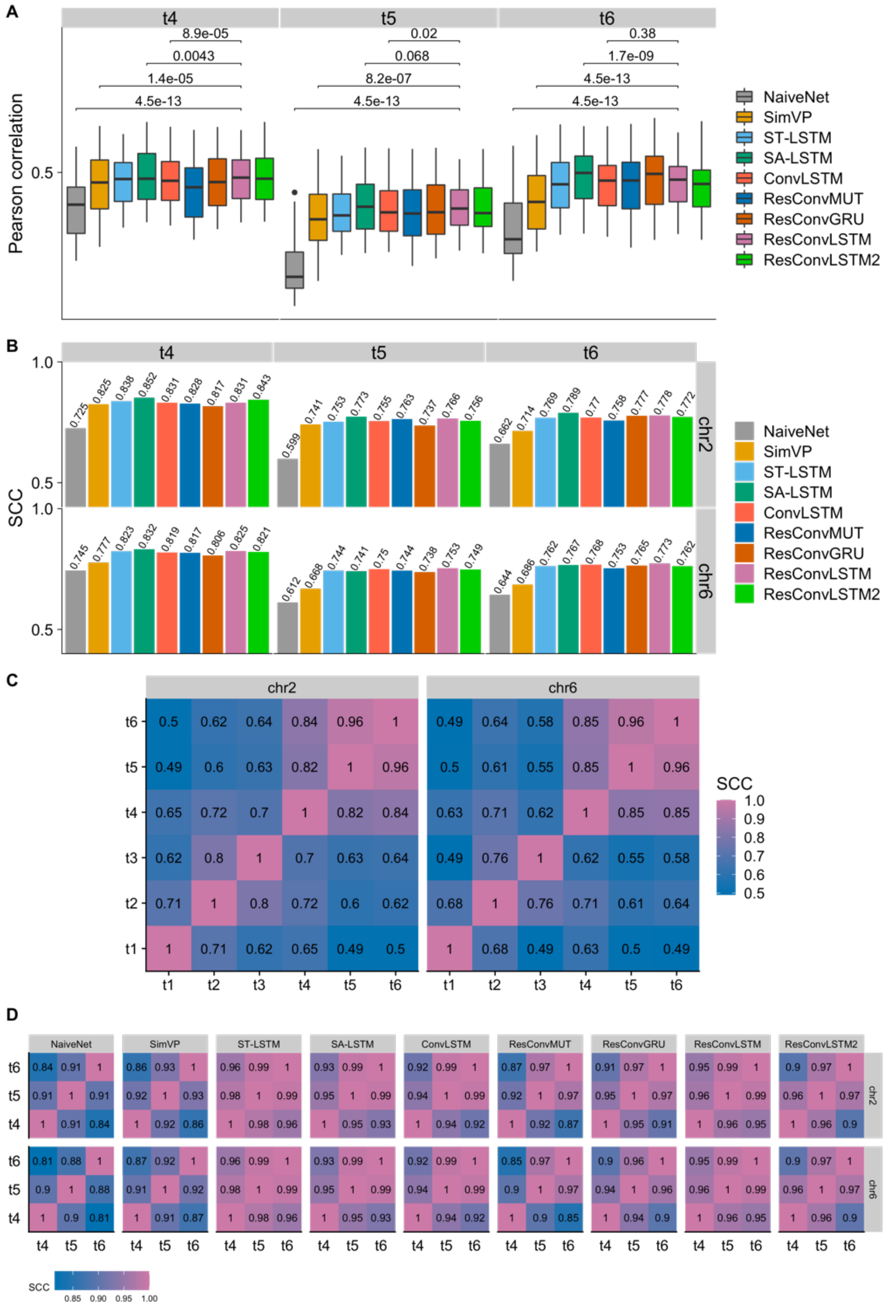

Fig. S13. Reproducibility results for dataset 3. (A) Boxplots of Pearson correlations between ground truth and predicted Hi-C contact matrices at each genomic distance for two testing chromosomes (2 and 6). For clarity, only P-values between ResConvLSTM and each of the other four methods (NaiveNet, SimVP, SA-LSTM, and ConvLSTM) were shown for all plots with statistical tests. P-values were computed using the paired Wilcoxon test. (B) SCC scores between ground truth and predicted Hi-C contact matrices from the nine methods. (C) SCC scores between ground-truth Hi-C matrices from each pair of the six time-steps for two testing chromosomes. (D) SCC scores between predicted Hi-C matrices from each pair of the three predicted time-steps.

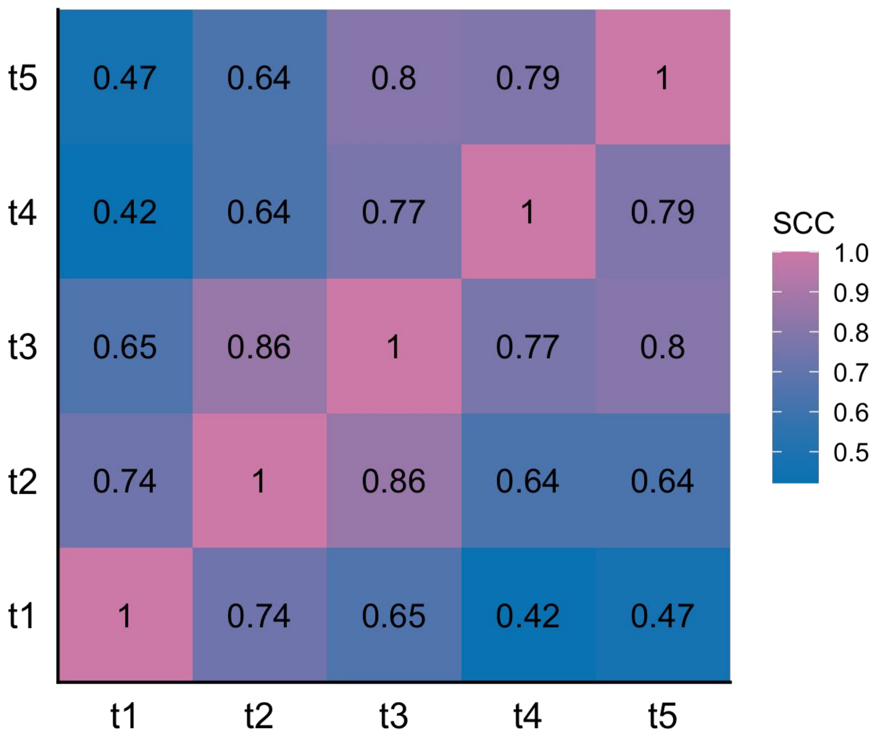

Fig. S14. Genome-wide average SCC scores between ground-truth Hi-C matrices from each pair of the five time-steps on dataset 4.

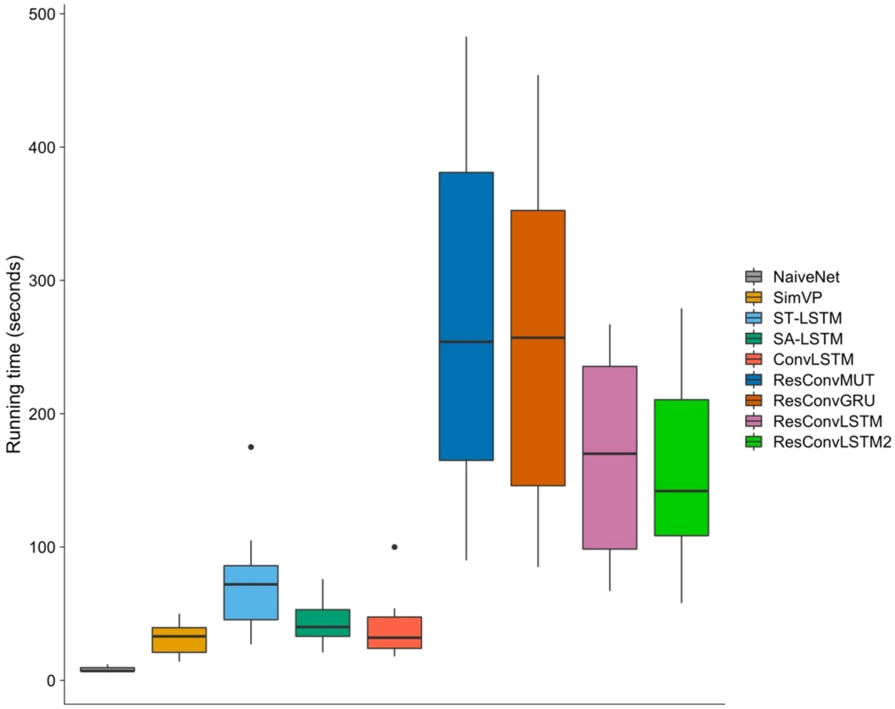

Fig. S15. Running time summary for predicting all chromosomes from dataset 4 when an NIVIDIA A100 equipped with 40GB memory was used and the batch size for all testing data is set to one. A minimum of 2.5 GB of GPU memory is required for all nine models.

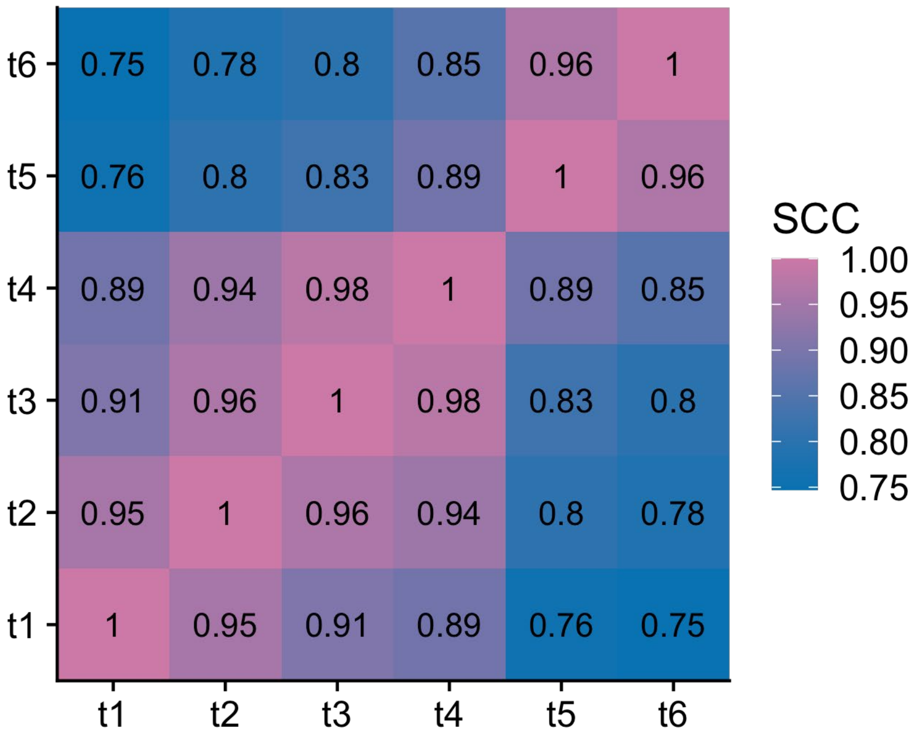

Fig. S16. Genome-wide average SCC scores between ground-truth Hi-C matrices from each pair of the six time-steps on dataset 5.

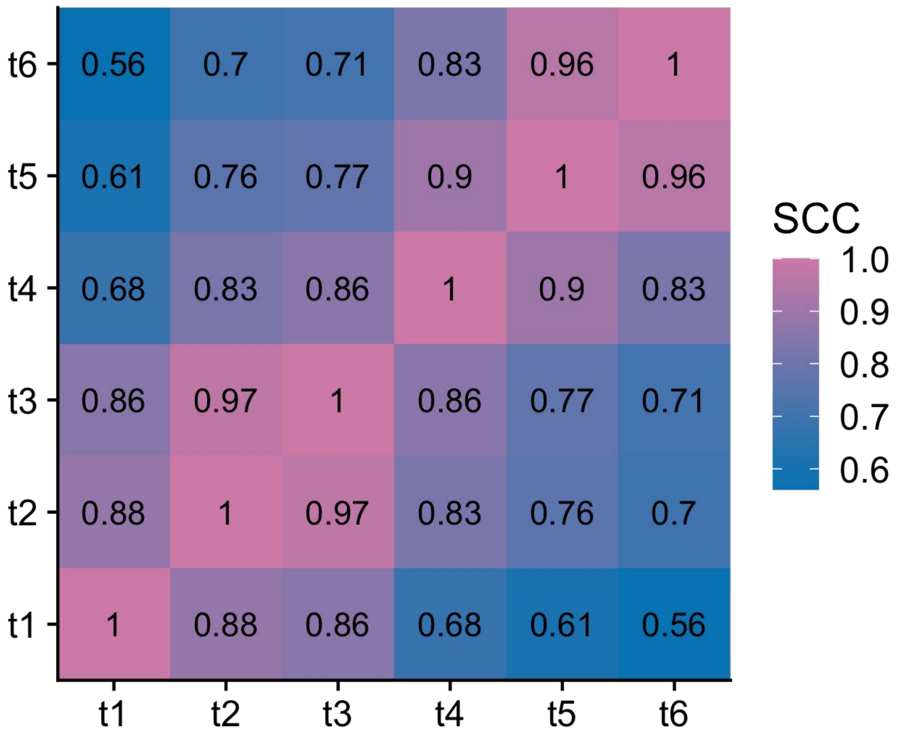

Fig. S17. Genome-wide average SCC scores between ground-truth Hi-C matrices from each pair of the six time-steps on dataset 6.

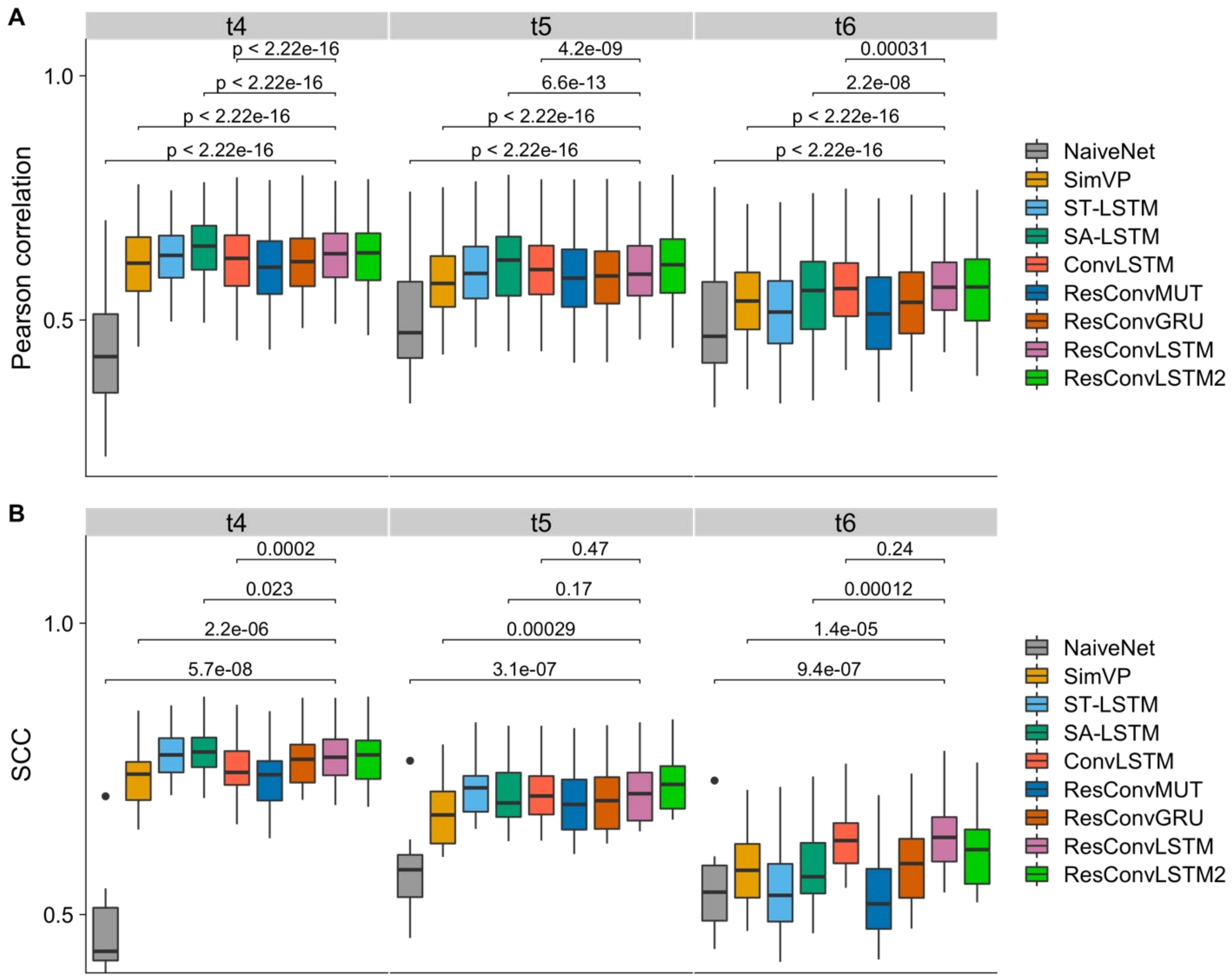

Fig. S18. Reproducibility results for dataset 6. (A) Boxplots of Pearson correlations at each genomic distance. P-values were computed using the paired Wilcoxon test. (B) SCC scores between ground truth and predicted Hi-C contact matrices. P-values were computed using the paired t-test. Both correlation and SCC values were collected from all chromosomes.

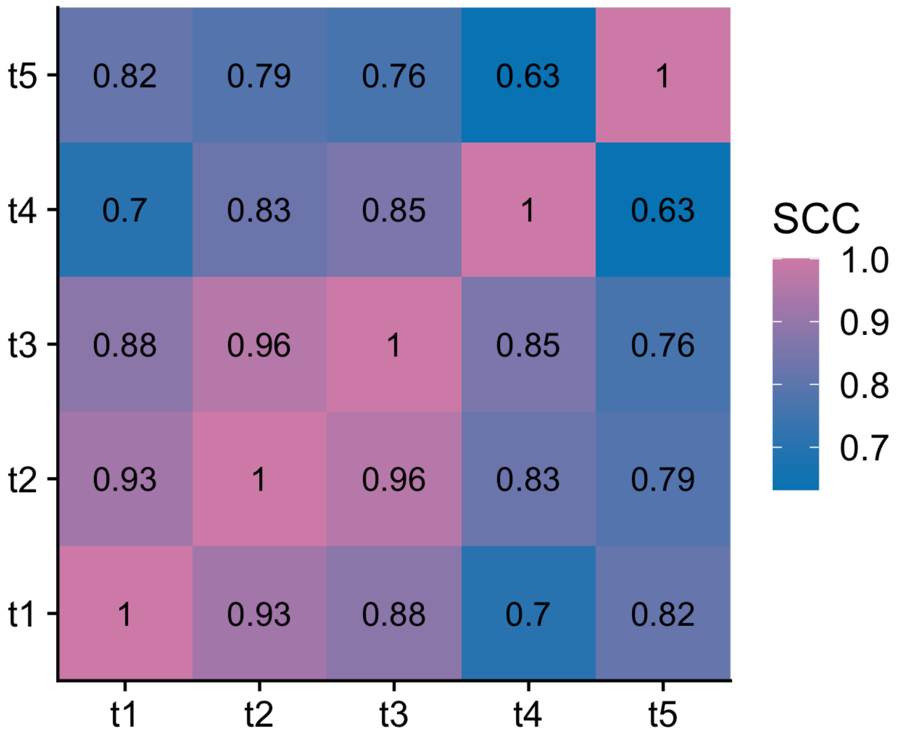

Fig. S19. Genome-wide average SCC scores between ground-truth Hi-C matrices from each pair of the five time-steps on dataset 7.

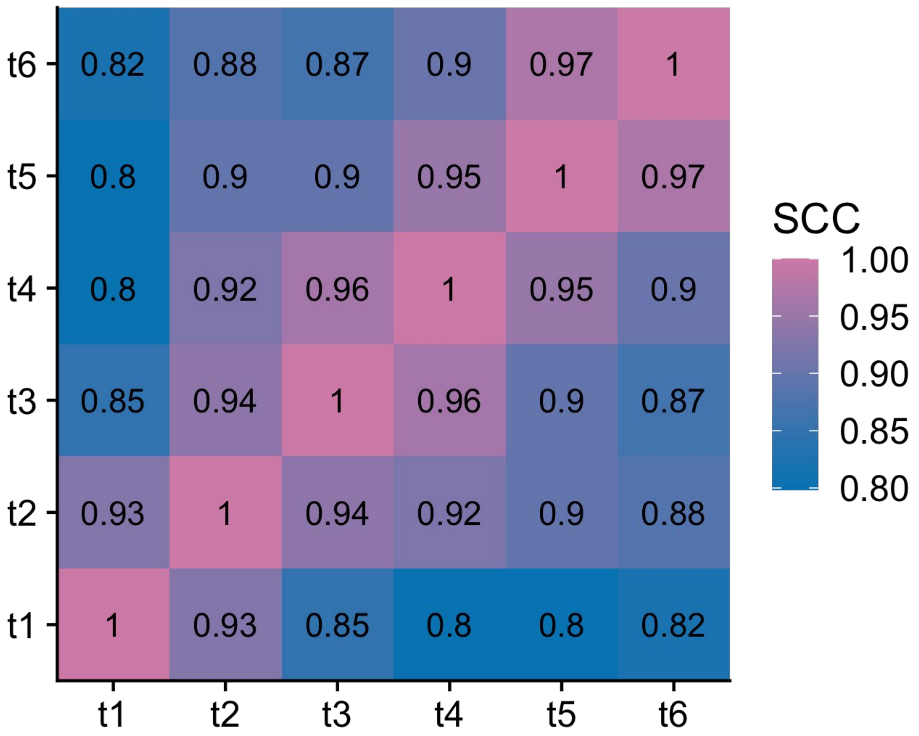

Fig. S20. Genome-wide average SCC scores between ground-truth Hi-C matrices from each pair of the six time-steps on dataset 8.

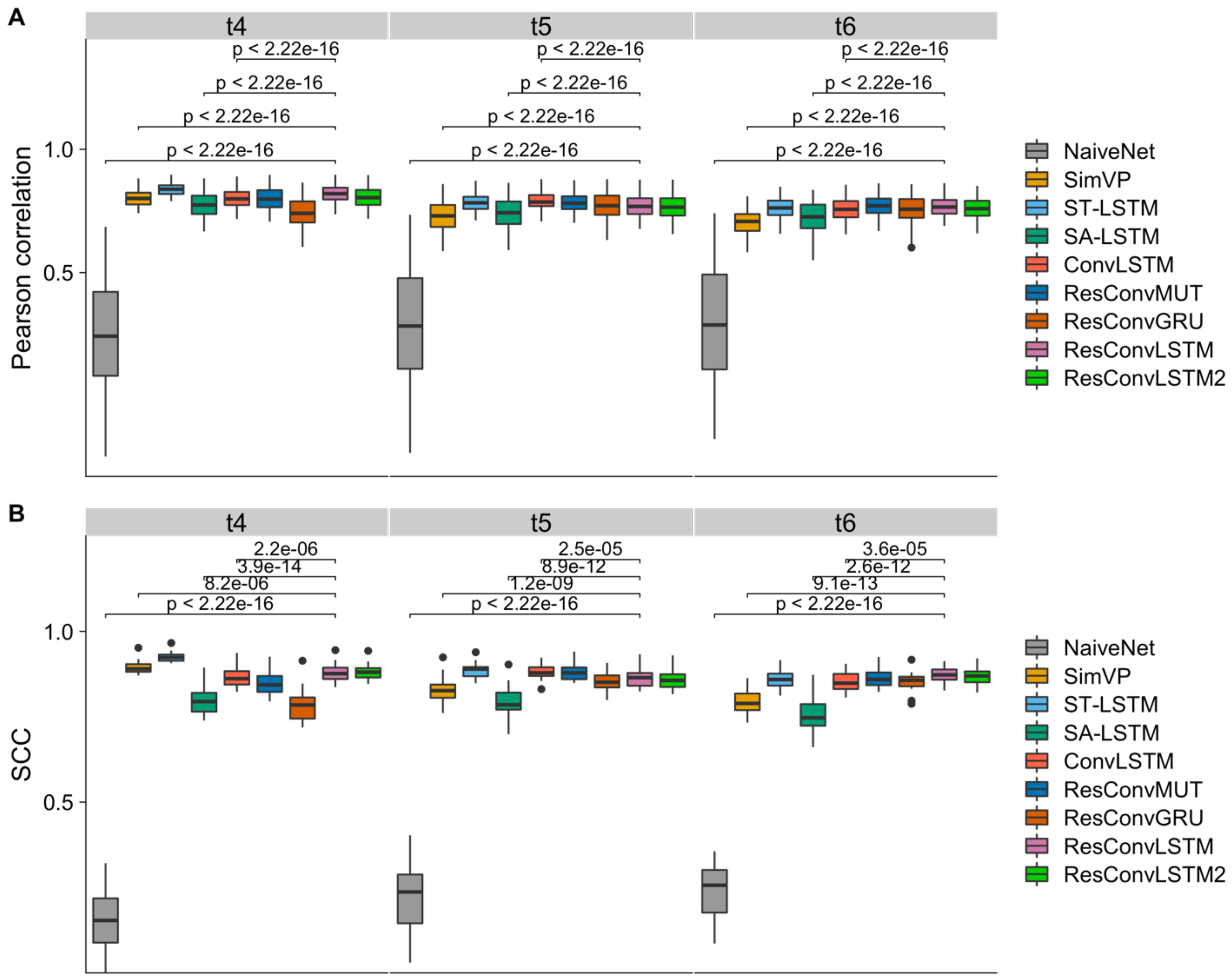

Fig. S21. Reproducibility results for dataset 8. (A) Boxplots of Pearson correlations at each genomic distance. P-values were computed using the paired Wilcoxon test. (B) SCC scores between ground truth and predicted Hi-C contact matrices. P-values were computed using the paired t-test. Both correlation and SCC values were collected from all chromosomes.
